# Plant Species Identification Using Computer Vision Techniques: A Systematic Literature Review

**DOI:** 10.1007/s11831-016-9206-z

**Published:** 2017-01-07

**Authors:** Jana Wäldchen, Patrick Mäder

**Affiliations:** 1 Department Biogeochemical Integration, Max Planck Institute for Biogeochemistry, Hans Knöll Strasse 10, 07745 Jena, Germany; 2 Software Engineering for Safety-Critical Systems, Technische Universität Ilmenau, Helmholtzplatz 5, 98693 Ilmenau, Germany

## Abstract

Species knowledge is essential for protecting biodiversity. The identification of plants by conventional keys is complex, time consuming, and due to the use of specific botanical terms frustrating for non-experts. This creates a hard to overcome hurdle for novices interested in acquiring species knowledge. Today, there is an increasing interest in automating the process of species identification. The availability and ubiquity of relevant technologies, such as, digital cameras and mobile devices, the remote access to databases, new techniques in image processing and pattern recognition let the idea of automated species identification become reality. This paper is the first systematic literature review with the aim of a thorough analysis and comparison of primary studies on computer vision approaches for plant species identification. We identified 120 peer-reviewed studies, selected through a multi-stage process, published in the last 10 years (2005–2015). After a careful analysis of these studies, we describe the applied methods categorized according to the studied plant organ, and the studied features, i.e., shape, texture, color, margin, and vein structure. Furthermore, we compare methods based on classification accuracy achieved on publicly available datasets. Our results are relevant to researches in ecology as well as computer vision for their ongoing research. The systematic and concise overview will also be helpful for beginners in those research fields, as they can use the comparable analyses of applied methods as a guide in this complex activity.

## Introduction

Biodiversity is declining steadily throughout the world [113]. The current rate of extinction is largely the result of direct and indirect human activities [95]. Building accurate knowledge of the identity and the geographic distribution of plants is essential for future biodiversity conservation [69]. Therefore, rapid and accurate plant identification is essential for effective study and management of biodiversity.

In a manual identification process, botanist use different plant characteristics as identification keys, which are examined sequentially and adaptively to identify plant species. In essence, a user of an identification key is answering a series of questions about one or more attributes of an unknown plant (e.g., shape, color, number of petals, existence of thorns or hairs) continuously focusing on the most discriminating characteristics and narrowing down the set of candidate species. This series of answered questions leads eventually to the desired species. However, the determination of plant species from field observation requires a substantial botanical expertise, which puts it beyond the reach of most nature enthusiasts. Traditional plant species identification is almost impossible for the general public and challenging even for professionals that deal with botanical problems daily, such as, conservationists, farmers, foresters, and landscape architects. Even for botanists themselves species identification is often a difficult task. The situation is further exacerbated by the increasing shortage of skilled taxonomists [47]. The declining and partly nonexistent taxonomic knowledge within the general public has been termed “taxonomic crisis” [35].

The still existing, but rapidly declining high biodiversity and a limited number of taxonomists represents significant challenges to the future of biological study and conservation. Recently, taxonomists started searching for more efficient methods to meet species identification requirements, such as developing digital image processing and pattern recognition techniques [47]. The rich development and ubiquity of relevant information technologies, such as digital cameras and portable devices, has brought these ideas closer to reality. Digital image processing refers to the use of algorithms and procedures for operations such as image enhancement, image compression, image analysis, mapping, and geo-referencing. The influence and impact of digital images on the modern society is tremendous and is considered a critical component in a variety of application areas including pattern recognition, computer vision, industrial automation, and healthcare industries [131].

Image-based methods are considered a promising approach for species identification [47, 69, 133]. A user can take a picture of a plant in the field with the build-in camera of a mobile device and analyze it with an installed recognition application to identify the species or at least to receive a list of possible species if a single match is impossible. By using a computer-aided plant identification system also non-professionals can take part in this process. Therefore, it is not surprising that large numbers of research studies are devoted to automate the plant species identification process. For instance, ImageCLEF, one of the foremost visual image retrieval campaigns, is hosting a plant identification challenge since 2011. We hypothesize that the interest will further grow in the foreseeable future due to the constant availability of portable devices incorporating myriad precise sensors. These devices provide the basis for more sophisticated ways of guiding and assisting people in species identification. Furthermore, approaching trends and technologies such as augmented reality, data glasses, and 3D-scans give this research topic a long-term perspective.

**Fig. 1:**
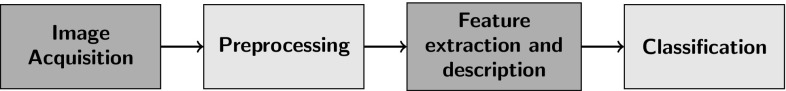
Generic steps of an image-based plant classification process (*green-shaded boxes* are the main focus of this review). (Color figure online)

An image classification process can generally be divided into the following steps (cp. Fig. 1):

- **Image acquisition**—The purpose of this step is to obtain the image of a whole plant or its organs so that analysis towards classification can be performed.
- **Preprocessing**—The aim of image preprocessing is enhancing image data so that undesired distortions are suppressed and image features that are relevant for further processing are emphasized. The preprocessing sub-process receives an image as input and generates a modified image as output, suitable for the next step, the feature extraction. Preprocessing typically includes operations like image denoising, image content enhancement, and segmentation. These can be applied in parallel or individually, and they may be performed several times until the quality of the image is satisfactory [51, 124].
- **Feature extraction and description**—Feature extraction refers to taking measurements, geometric or otherwise, of possibly segmented, meaningful regions in the image. Features are described by a set of numbers that characterize some property of the plant or the plant’s organs captured in the images (aka descriptors) [124].
- **Classification**—In the classification step, all extracted features are concatenated into a feature vector, which is then being classified.

The main objectives of this paper are (1) reviewing research done in the field of automated plant species identification using computer vision techniques, (2) to highlight challenges of research, and (3) to motivate greater efforts for solving a range of important, timely, and practical problems. More specifically, we focus on the *Image Acquisition* and the *Feature Extraction and Description* step of the discussed process since these are highly influenced by the object type to be classified, i.e., plant species. A detailed analysis of the *Preprocessing* and the *Classification* steps is beyond the possibilities of this review. Furthermore, the applied methods within these steps are more generic and mostly independent of the classified object type.

## Methods

We followed the methodology of a systematic literature review (SLR) to analyze published research in the field of automated plant species identification. Performing a SLR refers to assessing all available research concerning a research subject of interest and to interpret aggregated results of this work. The whole process of the SLR is divided into three fundamental steps: (I) defining research questions, (II) conducting the search process for relevant publications, and (III) extracting necessary data from identified publications to answer the research questions [75, 109].

### Research Questions

We defined the following five research questions:

*RQ-1:* *Data demographics: How are time of publication, venue, and geographical author location distributed across primary studies?*—The aim of this question is getting an quantitative overview of the studies and to get an overview about the research groups working on this topic.

*RQ-2:* *Image Acquisition: How many images of how many species were analyzed per primary study, how were these images been acquired, and in which context have they been taken?*—Given that the worldwide estimates of flowering plant species (aka angiosperms) vary between 220,000 [90, 125] and 420,000 [52], we would like to know how many species were considered in studies to gain an understanding of the generalizability of results. Furthermore, we are interested in information on where plant material was collected (e.g., fresh material or web images); and whether the whole plant was studied or selected organs.

*RQ-3:* *Feature detection and extraction: Which features were extracted and which techniques were used for feature detection and description?*—The aim of this question is categorizing, comparing, and discussing methods for detecting and describing features used in automated plant species classification.

*RQ-4:* *Comparison of studies: Which methods yield the best classification accuracy?*—To answer this question, we compare the results of selected primary studies that evaluate their methods on benchmark datasets. The aim of this question is giving an overview of utilized descriptor-classifier combinations and the achieved accuracies in the species identification task.

*RQ-5:* *Prototypical implementation: Is a prototypical implementation of the approach such as a mobile app, a web service, or a desktop application available for evaluation and actual usage?*—This question aims to analyzes how ready approaches are to be used by a larger audience, e.g., the general public.

### Data Sources and Selection Strategy

**Fig. 2:**
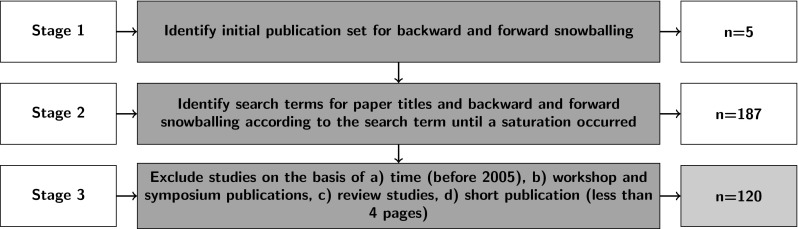
Study selection process

We used a combined backward and forward snowballing strategy for the identification of primary studies (see Fig. 2). This search technique ensures to accumulate a relatively complete census of relevant literature not confined to one research methodology, one set of journals and conferences, or one geographic region. Snowballing requires a starting set of publications, which should either be published in leading journals of the research area or have been cited many times. We identified our starting set of five studies through a manual search on Google Scholar (see Table 1). Google Scholar is a good alternative to avoid bias in favor of a specific publisher in the initial set of the sampling procedure. We then checked whether the publications in the initial set were included in at least one of the following scientific repositories: (a) Thomson Reuters Web of Science^TM^, (b) IEEE Xplore^®^, (c) ACM Digital Library, and (d) Elsevier ScienceDirect^®^. Each publication identified in any of the following steps was also checked for being listed in at least one of these repositories to restrict our focus to high quality publications solely.

**Table 1:** Seeding set of papers for the backward and forward snowballing

Study	Journal	Topic	Year	$\sum$ Refs.	$\sum$ Cits.
Gaston and O'Neill [47]	Philosophical Transactions of the Royal Society of London	Roadmap paper on automated species identification	2004	91	215
MacLeod et al. [88]	Nature	Roadmap paper on automated species identification	2010	10	104
Cope et al. [33]	Expert Systems with Applications	Review paper on automated leaf identification	2012	113	108
Nilsback et al. [105]	Indian Conference on Computer Vision, Graphics and Image Processing	Study paper on automated flower recognition	2008	18	375
Du et al. [40]	Applied Mathematics and Computation	Study paper on automated leaf recognition	2007	20	215

Table notes: Number of citations based on Google Scholar, accessed June 2016

Backward snowball selection means that we recursively considered the referenced publications in each paper derived through manual search as candidates for our review. Forward snowballing analogously means that we, based on Google Scholar citations, identified additional candidate publications from all those studies that were citing an already included publication. For a candidate to be included in our study, we checked further criteria in addition to being listed in the four repositories. The criteria referred to the paper title, which had to comply to the following pattern:

**S1 AND (S2 OR S3 OR S4 OR S5 OR S6) AND NOT (S7) where**

- S1: (plant* OR flower* OR leaf OR leaves OR botan*)
- S2: (recognition OR recognize OR recognizing OR recognized)
- S3: (identification OR identify OR identifying OR identified)
- S4: (classification OR classify OR classifying OR classified)
- S5: (retrieval OR retrieve OR retrieving OR retrieved)
- S6: (“image processing” OR “computer vision”)
- S7: (genetic OR disease* OR “remote sensing” OR gene OR DNA OR RNA).

Using this search string allowed us to handle the large amount of existing work and ensured to search for primary studies focusing mainly on plant identification using computer vision. The next step, was removing studies from the list that had already been examined in a previous backward or forward snowballing iteration. The third step, was removing all studies that were not listed in the four literature repositories listed before. The remaining studies became candidates for our survey and were used for further backward and forward snowballing. Once no new papers were found, neither through backward nor through forward snowballing, the search process was terminated. By this selection process we obtained a candidate list of 187 primary studies.

To consider only high quality peer reviewed papers, we eventually excluded all workshop and symposium papers as well as working notes and short papers with less than four pages. Review papers were also excluded as they constitute no primary studies. To get an overview of the more recent research in the research area, we restricted our focus to the last 10 years and accordingly only included papers published between 2005 and 2015. Eventually, the results presented in this SLR are based upon 120 primary studies complying to all our criteria.

### Data Extraction

To answer RQ-1, corresponding information was extracted mostly from the meta-data of the primary studies. Table 2 shows that the data extracted for addressing RQ-2, RQ-3, RQ-4, and RQ-5 are related to the methodology proposed by a specific study. We carefully analyzed all primary studies and extracted necessary data. We designed a data extraction template used to collect the information in a structured manner (see Table 2). The first author of this review extracted the data and filled them into the template. The second author double-checked all extracted information. The checker discussed disagreements with the extractor. If they failed to reach a consensus, other researchers have been involved to discuss and resolve the disagreements.

**Table 2:** Simplified overview of the data extraction template

*RQ-1*	
Study identifier	
Year of publication	[2005–2015]
Country of all author(s)	
Authors’ background	[Biology/Ecology, Computer science/Engineering, Education]
Publication type	[journal, conference proceedings]
*RQ-2*	
Depicted organ(s)$^{\mathrm{a}}$	[leaf, flower, fruit, stem, whole plant]
No. of species	
No. of images	
Image source(s)$^{\mathrm{a}}$	[own dataset, existing dataset]
(a) own dataset	[fresh material, herbarium specimen, web]
(b) existing dataset	[name, no. of species, no. of images, source]
Image type$^{\mathrm{a}}$	[photo, scan, pseudo-scan]
Image background$^{\mathrm{a}}$	[natural, plain]
Considering:	
(a) damaged leaves	[yes, no]
(b) overlapped leaves	[yes, no]
(c) compound leaves	[yes, no]
*RQ-3*	
Studied organ$^{\mathrm{a}}$	[leaf, flower, fruit, stem, whole plant]
Studied feature(s)$^{\mathrm{a}}$	[shape, color, texture, margin, vein]
Studied descriptor(s)$^{\mathrm{a}}$	
*RQ-4*	
Utilized dataset	
No. of species	
Studied feature(s)$^{\mathrm{a}}$	
Applied classifier	
Achieved accuracy	
*RQ-5*	
Prototype name	
Type of application	[mobile, web, desktop]
Computation$^{\mathrm{a}}$	[online, offline]
Publicly available	[yes, no]
Supported organ$^{\mathrm{a}}$	[leaf, flower, multi-organ]
Expected background$^{\mathrm{a}}$	[plain, natural]

^a^Multiple values possible

### Threats to Validity

The main threats to the validity of this review stem from the following two aspects: study selection bias and possible inaccuracy in data extraction and analysis. The selection of studies depends on the search strategy, the literature sources, the selection criteria, and the quality criteria. As suggested by [109], we used multiple databases for our literature search and provide a clear documentation of the applied search strategy enabling replication of the search at a later stage. Our search strategy included a filter on the publication title in an early step. We used a predefined search string, which ensures that we only search for primary studies that have the main focus on plant species identification using computer vision. Therefore, studies that propose novel computer vision methods in general and evaluating their approach on a plant species identification task as well as studies that used unusual terminology in the publication title may have been excluded by this filter. Furthermore, we have limited ourselves to English-language studies. These studies are only journal and conference papers with a minimum of four pages. However, this strategy excluded non-English papers in national journals and conferences. Furthermore, inclusion of grey literature such as PhD or master theses, technical reports, working notes, and white-papers also workshop and symposium papers might have led to more exhaustive results. Therefore, we may have missed relevant papers. However, the ample list of included studies indicates the width of our search. In addition, workshop papers as well as grey literature is usually finally published on conferences or in journals. Therefore excluding grey literature and workshop papers avoids duplicated primary studies within a literature review. To reduce the threat of inaccurate data extraction, we elaborated a specialized template for data extraction. In addition, all disagreements between extractor and checker of the data were carefully considered and resolved by discussion among the researchers.

## Results

This section reports aggregated results per research question based on the data extracted from primary studies.

### Data Demographics (RQ-1)

**Fig. 3:**
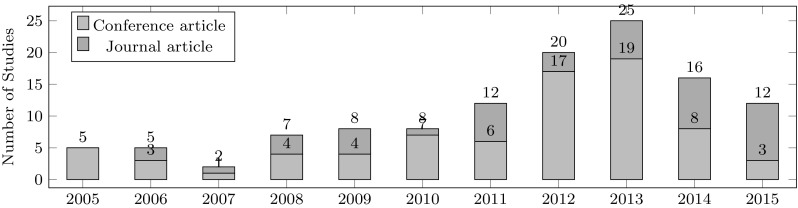
Number of studies per year of publication

To study the relative interest in automating plant identification over time, we aggregated paper numbers by year of publication (see Fig. 3). The figure shows a continuously increasing interest in this research topic. Especially, the progressively rising numbers of published papers in recent years show that this research topic is considered highly relevant by researchers today.

To gain an overview of active research groups and their geographical distribution, we analyzed the first author’s affiliation. The results depict that the selected papers are written by researchers from 25 different countries. More than half of these papers are from Asian countries (73/120), followed by European countries (26/120), American countries (14/120), Australia (4/120), and African countries (3/120). 34 papers have a first author from China, followed by France (17), and India (13). 15 papers are authored by a group located in two or more different countries. 108 out of the 120 papers are written solely by researches with computer science or engineering background. Only one paper is solely written by an ecologist. Ten papers are written in interdisciplinary groups with researchers from both fields. One paper was written in an interdisciplinary group where the first author has an educational and the second author an engineering background.

### Image Acquisition (RQ-2)

The purpose of this first step within the classification process is obtaining an image of the whole plant or its organs for later analysis towards plant classification.

#### Studied Plant Organs

Identifying species requires recognizing one or more characteristics of a plant and linking them with a name, either a common or so-called scientific name. Humans typically use one or more of the following characteristics: the plant as a whole (size, shape, etc.), its flowers (color, size, growing position, inflorescence, etc.), its stem (shape, node, outer character, bark pattern, etc.), its fruits (size, color, quality, etc.), and its leaves (shape, margin, pattern, texture, vein etc.) [114].

**Fig. 4:**
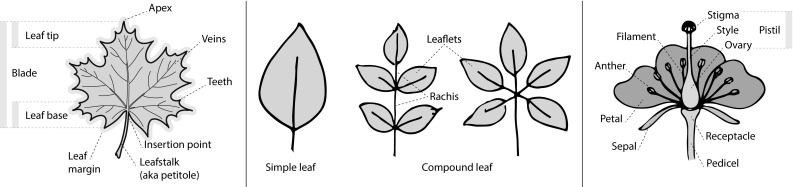
Leaf structure, leaf types, and flower structure

A majority of primary studies utilizes leaves for discrimination (106 studies). In botany, a leaf is defined as a usually green, flattened, lateral structure attached to a stem and functioning as a principal organ of photosynthesis and transpiration in most plants. It is one of the parts of a plant which collectively constitutes its foliage [44, 123]. Figure 4 shows the main characteristics of leaves with their corresponding botanical terms. Typically, a leaf consists of a blade (i.e., the flat part of a leaf) supported upon a petiole (i.e., the small stalk situated at the lower part of the leaf that joins the blade to the stem), which, continued through the blade as the midrib, gives off woody ribs and veins supporting the cellular texture. A leaf is termed “simple” if its blade is undivided, otherwise it is termed “compound” (i.e., divided into two or more leaflets). Leaflets may be arranged on either side of the rachis in pinnately compound leaves and centered around the base point (the point that joins the blade to the petiole) in palmately compound leaves [44]. Most studies use simple leaves for identification, while 29 studies considered compound leaves in their experiments. The internal shape of the blade is characterized by the presence of vascular tissue called veins, while the global shape can be divided into three main parts: (1) the leaf base, usually the lower 25% of the blade; the insertion point or base point, which is the point that joins the blade to the petiole, situated at its center. (2) The leaf tip, usually the upper 25% of the blade and centered by a sharp point called the apex. (3) The margin, which is the edge of the blade [44]. These local leaf characteristics are often used by botanists in the manual identification task and could also be utilized for an automated classification. However, the majority of existing leaf classification approaches rely on global leaf characteristics, thus ignoring these local information of leaf characteristics. Only eight primary studies consider local characteristics of leaves like the petiole, blade, base, and apex for their research [19, 85, 96, 97, 99, 119, 120, 158]. The characteristics of the leave margin is studied by six primary studies [18, 21, 31, 66, 85, 93].

In contrast to studies on leaves or plant foliage, a smaller number of 13 primary studies identify species solely based on flowers [3, 29, 30, 57, 60, 64, 104, 105, 112, 117, 128, 129, 149]. Some studies did not only focus on the flower region as a whole but also on parts of the flower. Hsu et al. [60] analyzed the color and shape not only of the whole flower region but also of the pistil area. Tan et al. [128] studied the shape of blooming flowers’ petals and [3] proposed analyzing the lip (labellum) region of orchid species. Nilsback and Zisserman [104, 105] propose features, which capture color, texture, and shape of petals as well as their arrangement.

Only one study proposes a multi-organ classification approach [68]. Contrary to other approaches that analyze a single organ captured in one image, their approach analyzes up to five different plant views capturing one or more organs of a plant. These different views are: full plant, flower, leaf (and leaf scan), fruit, and bark. This approach is the only one in this review dealing with multiple images exposing different views of a plant.

#### Images: Categories and Datasets

Utilized images in the studies fall into three categories: scans, pseudo-scans, and photos. While scan and pseudo-scan categories correspond respectively to plant images obtained through scanning and photography in front of a simple background, the photo category corresponds to plants photographed on natural background [49]. The majority of utilized images in the primary studies are scans and pseudo-scans thereby avoiding to deal with occlusions and overlaps (see Table 3). Only 25 studies used photos that were taken in a natural environment with cluttered backgrounds and reflecting a real-world scenario.

**Table 3:** Overview of utilized image data

Organ	Background	Image category	Studies	$\sum$
Leaf	Plain	Scans	[6–8, 14, 15, 17, 22, 25, 36, 37, 54, 62, 65, 78–80, 97–99, 106, 122, 145, 155]	23
		Pseudo-scans	[11, 26, 27, 32, 39, 41, 43, 46, 66, 67, 72, 76, 82, 118, 141, 156–159]	19
		Scans + pseudo-scans	[1, 4, 5, 16, 21, 23, 24, 28, 40, 48, 53, 56, 58, 59, 73, 77, 81, 87, 89, 91–94, 96, 103, 111, 114–116, 119, 121, 132–136, 139, 140, 143, 144, 146, 147, 150, 154]	43
		Illustrated leaf images	[100, 101, 107, 108]	4
		[No information]	[10, 31, 38, 42, 45, 110]	6
	Natural	Photos	[74, 102, 130]	3
	Plain + natural	Scans + pseudo-scans + photos	[18–20, 68, 85, 120, 137, 138, 148]	9
Flower	Natural	Photos	[3, 29, 30, 57, 60, 64, 68, 104, 105, 112, 117, 128, 129, 149]	14
Stem, fruit, full plant	Natural	Photos	[68]	1

Existing datasets of leaf images were uses in 62 primary studies. The most important (by usage) and publicly available datasets are:

- **Swedish leaf dataset**—The Swedish leaf dataset has been captured as part of a joined leaf classification project between the Linkoping University and the Swedish Museum of Natural History [127]. The dataset contains images of isolated leaf scans on plain background of 15 Swedish tree species, with 75 leaves per species (1125 images in total). This dataset is considered very challenging due to its high inter-species similarity [127]. The dataset can be downloaded here: http://www.cvl.isy.liu.se/en/research/datasets/swedish-leaf/.
- **Flavia dataset**—This dataset contains 1907 leaf images of 32 different species and 50–77 images per species. Those leaves were sampled on the campus of the Nanjing University and the Sun Yat-Sen arboretum, Nanking, China. Most of them are common plants of the Yangtze Delta, China [144]. The leaf images were acquired by scanners or digital cameras on plain background. The isolated leaf images contain blades only, without petioles (http://flavia.sourceforge.net/).
- **ImageCLEF11 and ImageCLEF12 leaf dataset**—This dataset contains 71 tree species of the French Mediterranean area captured in 2011 and further increased to 126 species in 2012. ImageCLEF11 contains 6436 pictures subdivided into three different groups of pictures: scans (48%), scan-like photos or pseudo-scans (14%), and natural photos (38%). The ImageCLEF12 dataset consists of 11,572 images subdivided into: scans (57%), scan-like photos (24%), and natural photos (19%). Both sets can be downloaded from ImageCLEF (2011) and ImageCLEF (2012): http://www.imageclef.org/.
- **Leafsnap dataset**—The Leafsnap dataset contains leave images of 185 tree species from the Northeastern United States. The images are acquired from two sources and are accompanied by automatically-generated segmentation data. The first source are 23,147 high-quality lab images of pressed leaves from the Smithsonian collection. These images appear in controlled backlit and front-lit versions, with several samples per species. The second source are 7719 field images taken with mobile devices (mostly iPhones) in outdoor environments. These images vary considerably in sharpness, noise, illumination patterns, shadows, etc. The dataset can be downloaded at: http://leafsnap.com/dataset/.
- **ICL dataset**—The ICL dataset contains isolated leaf images of 220 plant species with individual images per species ranging from 26 to 1078 (17,032 images in total). The leaves were collected at Hefei Botanical Garden in Hefei, the capital of the Chinese Anhui province by people from the local Intelligent Computing Laboratory (ICL) at the Institute of Intelligent Machines, China (http://www.intelengine.cn/English/dataset). All the leafstalks have been cut off before the leaves were scanned or photographed on a plain background.
- **Oxford Flower 17 and 102 datasets**—Nilsback and Zisserman [104, 105] have created two flower datasets by gathering images from various websites, with some supplementary images taken from their own photographs. Images show species in their natural habitat. The Oxford Flower 17 dataset consists of 17 flower species represented by 80 images each. The dataset contains species that have a very unique visual appearance as well as species with very similar appearance. Images exhibit large variations in viewpoint, scale, and illumination. The flower categories are deliberately chosen to have some ambiguity on each aspect. For example, some classes cannot be distinguished by color alone, others cannot be distinguished by shape alone. The Oxford Flower 102 dataset is larger than the Oxford Flower 17 and consists of 8189 images divided into 102 flower classes. The species chosen consist of flowers commonly occurring in the United Kingdom. Each class consists of between 40 and 258 images. The images are rescaled so that the smallest dimension is 500 pixels. The Oxford Flower 17 dataset is not a full subset of the 102 dataset neither in images nor in species. Both datasets can be downloaded at: http://www.robots.ox.ac.uk/ ~vgg/data/flowers/.

**Table 4:** Overview of utilized image datasets

Organ	Dataset		Studies	$\sum$
Leaf	Own dataset	Self-collected (imaged in lab)	[1, 5–8, 10, 11, 14, 15, 17, 26–28, 36–40, 53, 54, 56, 65–67, 72, 78, 79, 82, 89, 102, 114, 115, 118, 122, 130, 132, 134, 137, 138, 141, 144, 150, 154, 155, 158, 159]	46
		Web	[74, 138]	2
	Existing dataset	ImageCLEF11/ImageCLEF12	[4, 18–22, 85, 87, 91–94, 97–99, 119, 120, 135, 146, 148]	20
		Swedish leaf	[25, 62, 94, 119–121, 134–136, 145, 147, 158]	12
		ICL	[1, 62, 121, 135, 136, 139, 140, 145, 147, 156–158]	12
		Flavia	[1, 5, 16, 23, 24, 48, 58, 59, 73, 77, 81, 92, 94, 103, 111, 116, 120, 140, 144]	19
		Leafsnap	[56, 73, 96, 119, 120, 158]	6
		FCA	[48]	1
		Korea Plant Picture Book	[107, 108]	2
		Middle European Woody Plants (MEW)	[106]	1
		Southern China Botanical Garden	[143]	1
		Tela Database	[96]	1
	[No information]		[31, 32, 42, 45, 76, 110]	6
Flower	Own dataset	Self-collected (imaged in field)	[57]	1
		Self-collected (imaged in field) + web	[3, 29, 60, 104, 105, 112]	6
	Existing dataset	Oxford 17, Oxford 102	[117, 149]	3
	[No information]		[30, 64, 128, 129]	4
Flower, leaf, bark, fruit, full plant	Existing dataset	Social image collection	[68]	1

Forty-eight authors use their own, not publicly available, leaf datasets. For these leave images, typically fresh material was collected and photographed or scanned in the lab on plain background. Due to the great effort in collecting material, such datasets are limited both in the number of species and in the number of images per species. Two studies used a combination of self-collected leaf images and images from web resources [74, 138]. Most plant classification approaches only focus on intact plant organs and are not applicable to degraded organs (e.g., deformed, partial, or overlapped) largely existing in nature. Only 21 studies proposed identification approaches that can also handle damaged leaves [24, 38, 46, 48, 56, 58, 74, 93, 102, 132, 141, 143] and overlapped leaves [18–20, 38, 46, 48, 74, 85, 102, 122, 130, 137, 138, 148].

Most utilized flower images were taken by the authors themselves or acquired from web resources [3, 29, 60, 104, 105, 112]. Only one study solely used self-taken photos for flower analysis [57]. Two studies analyzed the Oxford 17 and the Oxford 102 datasets (Table 4).

**Fig. 5:**
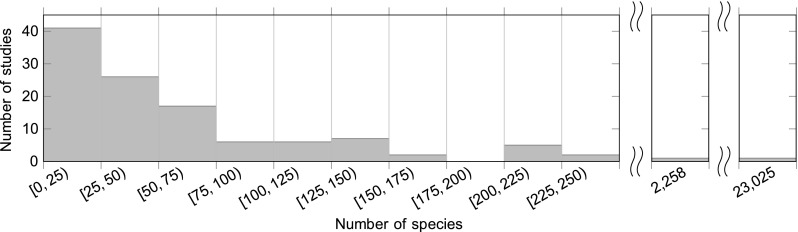
Distribution of the maximum evaluated species number per study. Six studies [76, 100, 101, 107, 108, 112] provide no information about the number of studied species. If more than one dataset per paper was used, species numbers refer to the largest dataset evaluated

**Fig. 6:**
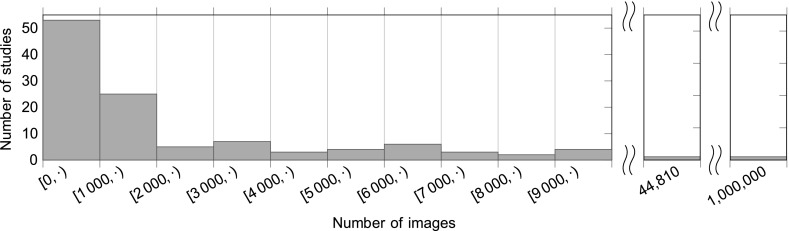
Distribution of the maximum evaluated images number per study. Six studies [10, 53, 76, 118, 132, 135] provide no information about the number of used images. If more than one dataset per paper was used, image numbers refer to the largest dataset evaluated

A majority of primary studies only evaluated their approach on datasets containing less than a hundred species (see Fig. 5) and at most a few thousand leaf images (see Fig. 6). Only two studies used a large dataset with more than 2000 species. Joly et al. [68] used a dataset with 2258 species and 44,810 images. In 2014 this was the plant identification study considering the largest number of species so far. In 2015 [143] published a study with 23,025 species represented by 1,000,000 images in total.

### Feature Detection and Extraction (RQ-3)

Feature extraction is the basis of content-based image classification and typically follows the preprocessing step in the classification process. A digital image is merely a collection of pixels represented as large matrices of integers corresponding to the intensities of colors at different positions in the image [51]. The general purpose of feature extraction is reducing the dimensionality of this information by extracting characteristic patterns. These patterns can be found in colors, textures and shapes [51]. Table 5 shows the studied features, separated for studies analyzing leaves and those analyzing flowers, and highlights that shape plays the most important role among the primary studies. 87 studies used leaf shape and 13 studies used flower shape for plant species identification. The texture of leaves and flowers is analyzed by 24 and 5 studies respectively. Color is mainly considered along with flower analysis (9 studies), but a few studies also used color for leaf analysis (5 studies). In addition, organ-specific features, i.e., leaf vein structure (16 studies) and leaf margin (8 studies), were investigated.

**Fig. 7:**
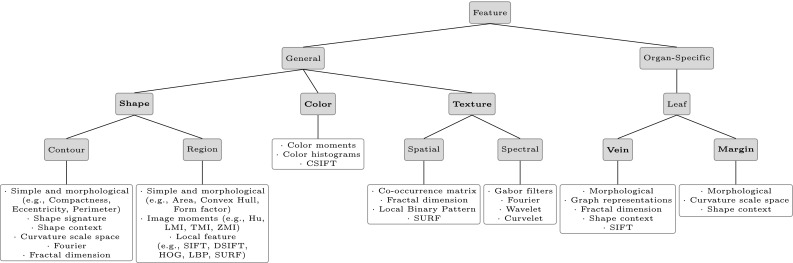
Categorization (*green shaded boxes*) and overview (*green framed boxes*) of the most prominent feature descriptors in plant species identification. Feature descriptors partly fall in multiple categories. (Color figure online)

Numerous methods exist in the literature for describing general and domain-specific features and new methods are being proposed regularly. Methods that were used for detecting and extracting features in the primary studies are highlighted in the subsequent sections. Because of perception subjectivity, there does not exist a single best presentation for a given feature. As we will see soon, for any given feature there exist multiple descriptions, which characterize the feature from different perspectives. Furthermore, different features or combinations of different features are often needed to distinguish different categories of plants. For example, whilst leaf shape may be sufficient to distinguish between some species, other species may have very similar leaf shapes to each other, but have different colored leaves or texture patterns. The same is also true for flowers. Flowers with the same color may differ in their shape or texture characteristics. Table 5 shows that 42 studies do not only consider one type of feature but use a combination of two or more feature types for describing leaves or flowers. No single feature may be sufficient to separate all the categories, making feature selection and description a challenging problem. Typically, this is the innovative part of the studies we reviewed. Segmentation and classification also allow for some flexibility, but much more limited. In the following sections, we will give an overview of the main features and their descriptors proposed for automated plant species classification (see also Fig. 7). First, we analyze the description of the general features starting with the most used feature shape, followed by texture, and color and later on we review the description of the organ-specific features leaf vein structure and leaf margin.

**Table 5:** Studied organs and features

Organ	Feature	Studies	$\sum$
Leaf	Shape	[1, 6, 11, 15, 19, 22, 24, 26, 28, 38–42, 45, 46, 54, 56, 58, 59, 62, 72, 76, 77, 81, 82, 89, 92, 94, 96–100, 102, 103, 106, 110, 111, 119–121, 130, 134, 135, 137, 138, 141, 145–147, 155–159]	56
	Texture	[7, 8, 17, 25, 32, 36, 37, 115, 118, 122, 132, 150]	12
	Margin	[31, 66]	2
	Vein	[53, 78–80]	4
	Shape + texture	[10, 23, 68, 91, 114, 136, 140, 143, 154]	9
	Shape + color	[16, 27, 87, 116]	4
	Shape + margin	[18, 20, 21, 73, 85, 93]	6
	Shape + vein	[4, 5, 14, 65, 67, 101, 107, 108, 139, 144]	10
	Shape + color + texture	[74, 148]	2
	Shape + color + texture + vein	[43, 48]	2
Flower	Shape	[64, 128, 129]	3
	Shape + color	[3, 30, 57, 60, 117]	5
	Shape + texture	[149]	1
	Shape + texture + color	[29, 68, 104, 105, 112]	5
Bark + fruit	Shape + texture	[68]	1
Full plant	Shape + texture + color	[68]	1

#### Shape

Shape is known as an important clue for humans when identifying real-world objects. A shape measure in general is a quantity, which relates to a particular shape characteristic of an object. An appropriate shape descriptor should be invariant to geometrical transformations, such as, rotation, reflection, scaling, and translation. A plethora of methods for shape representation can be found in the literature [151]. Shape descriptors are classified into two broad categories: contour-based and region-based. Contour-based shape descriptors extract shape features solely from the contour of a shape. In contrast, region-based shape descriptors obtain shape features from the whole region of a shape [72, 151]. In addition, there also exist some methods, which cannot be classified as either contour-based or region-based. In the following section, we restrict our discussion to those techniques that have been applied for plant species identification (see Table 6). We start our discussion with *simple and morphological shape descriptors (SMSD)* followed by a discussion of more sophisticated descriptors. Since the majority of studies focusses on plant identification via leaves, the discussed shape descriptors mostly apply to leaf shape classification. Techniques which were used for flower analysis will be emphasized.

**Table 6:** Studies analyzing the shape of organs solely or in combination with other features

Organ	Features	Shape descriptor	Studies
Leaf	**Shape**	SMSD	[24, 58, 82, 89, 141]
		SMSD, FD	[1]
		SMSD, moments (Hu)	[40, 110, 137]
		SMSD, moments (TMI), FD	[106]
		SMSD, moments (Hu, ZMI), FD	[72]
		SMSD, DFH	[146]
		Moments (Hu)	[102]
		Moments (Hu, ZMI)	[138]
		Moments (ZMI, LMI, TMI)	[159]
		FD	[147]
		CCD	[130]
		CCD, AC	[46]
		AT	[6]
		TAR, TSL, TOA, TSLA	[94]
		TAR, TSL, SC, salient points description	[92]
		CSS	[45]
		SMSD, CSS	[15]
		CSS, velocity representation	[28]
		HoCS	[76]
		SRVF	[77]
		IDSC	[11]
		I-IDSC, Gaussian shape pyramid	[158]
		MDM	[62]
		SIFT	[26, 59, 81]
		HOG	[41, 111, 145]
		HOG, central moments of order	[155]
		SURF	[103]
		Multi-scale overlapped block LBP	[121]
		MARCH	[134, 135]
		Describe leaf edge variation	[54]
		FD, procrustes analysis	[56]
		Polygonal approximation, invariant attributes sequence representation	[38, 39]
		Minimum perimeter polygons	[100]
		HOUGH, Fourier, EOH, LEOH, DFH	[99]
		Moments (Hu), centroid-Radii model, binary-Superposition	[22]
		Isomap, supervised isomap	[42]
		MLLDE algorithm	[156]
		MICA	[157]
		Parameters of the compound leaf model, parameters of the polygonal leaflet model, averaged parameters of base and apex models, averaged CSS-based contour parameters	[19]
		Leaf landmarks (leaf apex, the leaf base, centroid)	[119, 120]
		Detecting different leaf parts (local translational symmetry of small regions, local symmetry of depth indention)	[97]
		Detecting petitole shape (local translational symmetry of width)	[96]
		Geometric properties of local maxima and inflexion points	[98]
	**Shape**, color	SMSD	[16]
		SMSD, FD	[116]
		PHOG, Wavelet features	[87]
		SIFT	[27]
	**Shape**, margin	CSS, detecting teeth and pits	[18, 20, 21]
		SMSD, moments (Hu), MDM, AMD	[73]
		Advanced SC	[93]
	**Shape**, texture	SMSD, moments (Hu)	[154]
		CCD, AC	[10]
		CT, moments (Hu)	[23]
		Multi-resolution and multi-directional CT	[115]
		RSC	[114]
		CDS	[140]
		SC	[143]
		Advanced SC	[91]
		DS-LBP	[136]
		SURF, EOH, HOUGH	[68]
	**Shape**, vein	SMSD	[5, 144]
		RPWFF, FracDim, moments (Hu)	[65]
		FracDim	[14, 67]
		SC, SIFT	[139]
		Minimum perimeter polygons	[101, 107, 108]
		Contour covariance	[4]
	**Shape**, color, texture	SIFT, high curvature points on the contour	[74]
		SMSD, BSS, RMI, ACH, CPDH, FD	[148]
	**Shape**, color, texture, vein	SMSD	[43, 48]
Flower	**Shape**	SMSD	[129]
		Mathematical descriptor for petal shape	[128]
		Zero-crossing rate, the minimum distance, contour line’s length from the contour image	[64]
	**Shape**, color	Shape density distribution, edge density distribution	[30]
		SMSD, moments (Hu), FracDim, CCD	[3]
		SIFT, Dense SIFT, feature context	[117]
		CDD	[57]
		CDS, SMSD	[60]
	**Shape**, texture	SIFT	[149]
	**Shape**, color, texture	Edge densities, edge directions, moments (Hu)	[29]
		CSS	[112]
		SIFT	[104]
		SIFT, HOG	[105]
		SURF, EOH, HOUGH	[68]
Fruit, bark, full plant	**Shape**, texture	SURF, EOH, HOUGH	[68]

Abbreviations not explained in the text–*BSS* basic shape statistics, *CPDH* contour point distribution histogram, *CT* curvelet transform, *EOH* edge orientation histogram, *DFH* directional fragment histogram, *DS-LBP* dual-scale decomposition and local binary descriptors, *Fourier* Fourier histogram, *HOUGH* histogram of lines orientation and position, *LEOH* local edge orientation histogram, *MICA* multilinear independent component analysis, *MLLDE* modified locally linear discriminant embedding, *PHOG* pyramid histograms of oriented gradients, *RMI* regional moments of inertia, *RPWFF* ring projection wavelet fractal feature, *RSC* relative sub-image coefficients

#### Simple and Morphological Shape Descriptors

Across the studies we found six basic shape descriptors used for **leaf analysis** (see first six rows of Table 7). These refer to basic geometric properties of the leaf’s shape, i.e., diameter, major axis length, minor axis length, area, perimeter, centroid (see, e.g., [144]). On top of that, studies compute and utilize morphological descriptors based on these basic descriptors, e.g., aspect ratio, rectangularity measures, circularity measures, and the perimeter to area ratio (see Table 6). Table 6 shows that studies often employ ratios as shape descriptors. Ratios are simple to compute and naturally invariant to translation, rotation, and scaling; making them robust against different representations of the same object (aka leaf). In addition, several studies proposed more leaf-specific descriptors. For example, [58] introduce a *leaf width factor (LWF)*, which is extracted from leaves by slicing across the major axis and parallel to the minor axis. Then, the LWF per strip is calculated as the ratio of the width of the strip to the length of the entire leaf (major axis length). Yanikoglu et al. [148] propose an *area width factor (AWF)* constituting a slight variation of the LWF. For AWF, the area of each strip normalized by the global area is computed. As another example, [116] used a *porosity feature* to explain cracks in the leaf image (Table 7).

**Table 7:** Simple and morphological shape descriptors (SMSD)

Descriptor	Explanation	Pictogram	Formula	Studies	$\sum$
*Diameter* *D*	Longest distance between any two points on the margin of the organ	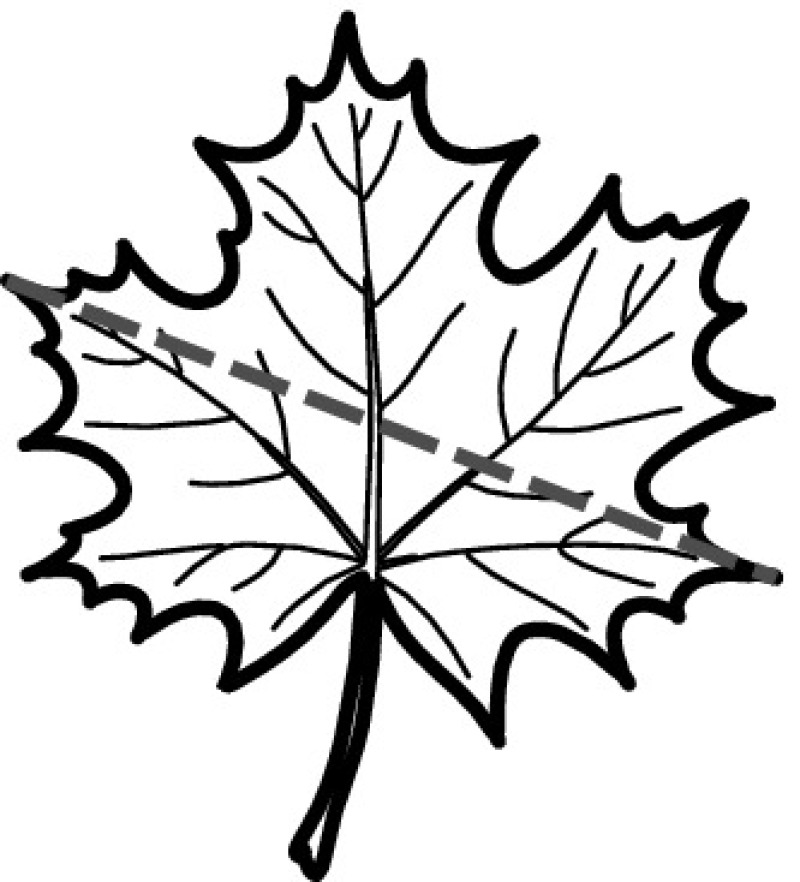		[5, 15, 16, 89, 110, 144]	6
*Major axis length* *L*	Line segment connecting the base and the tip of the leaf	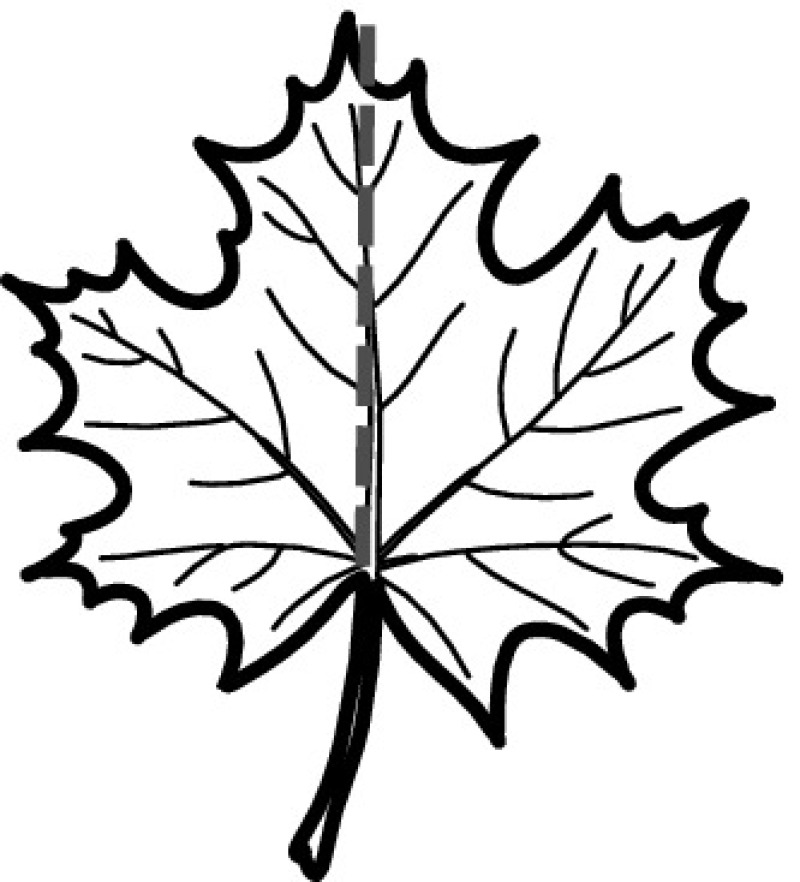		[5, 16, 24, 58, 89, 144]	6
*Minor axis length* *W*	Maximum width that is perpendicular to the *major axis*	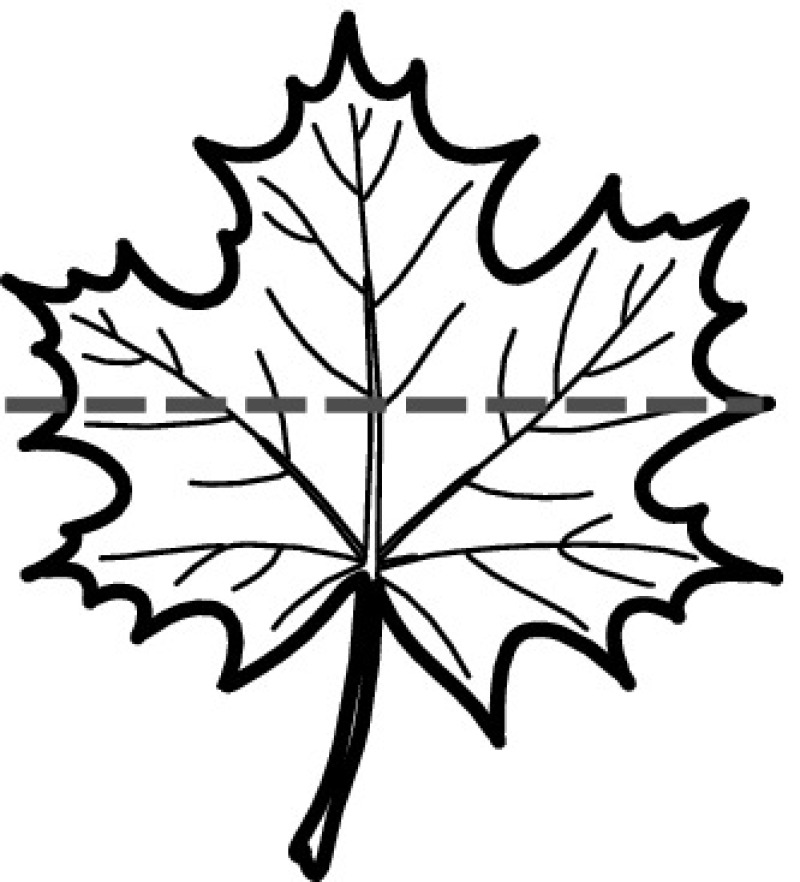		[5, 16, 24, 58, 89, 144]	6
*Area* *A*	Number of pixels in the region of the organ	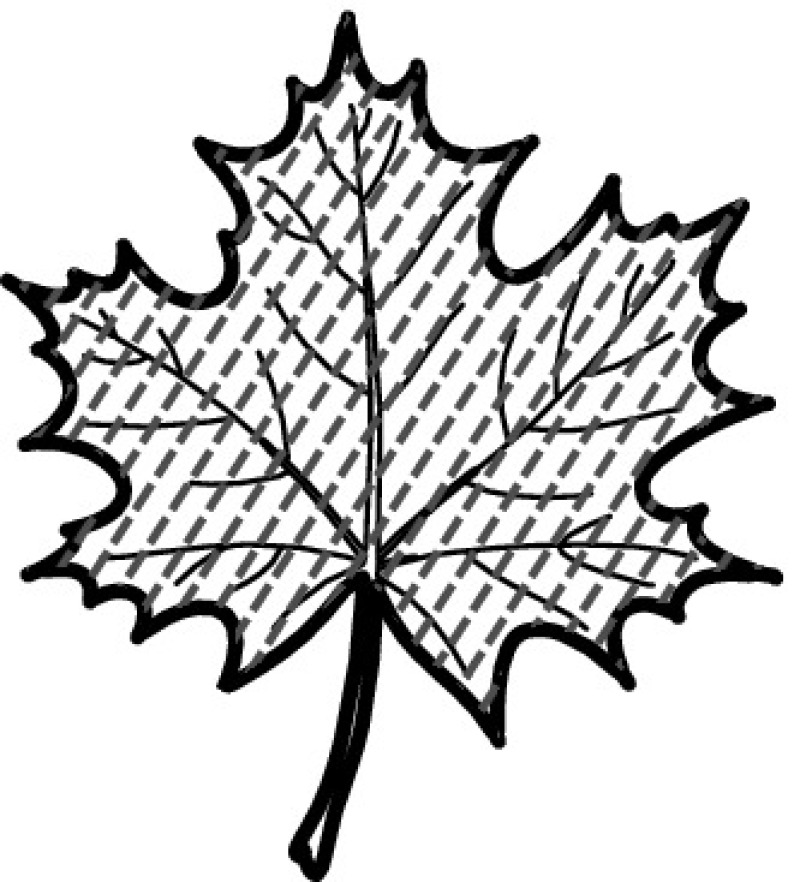		[5, 15, 16, 24, 58, 89, 129, 144]	8
*Perimeter* *P*	Summation of the distances between each adjoining pair of pixels around the border of the organ	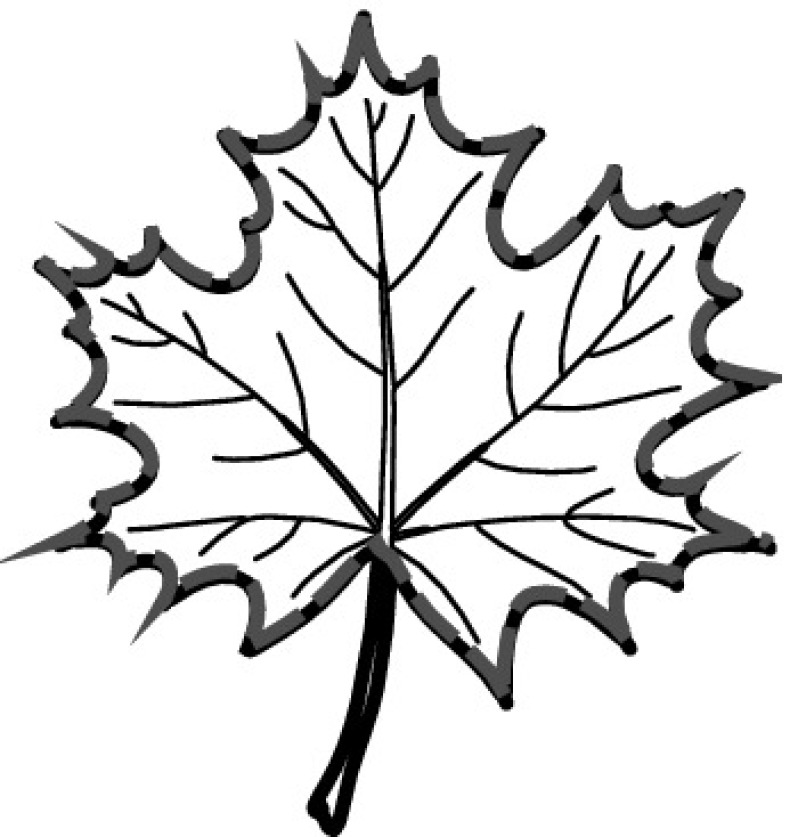		[5, 16, 24, 48, 58, 89, 129, 144]	8
*Centroid*	Represents the coordinates of the organ’s geometric center	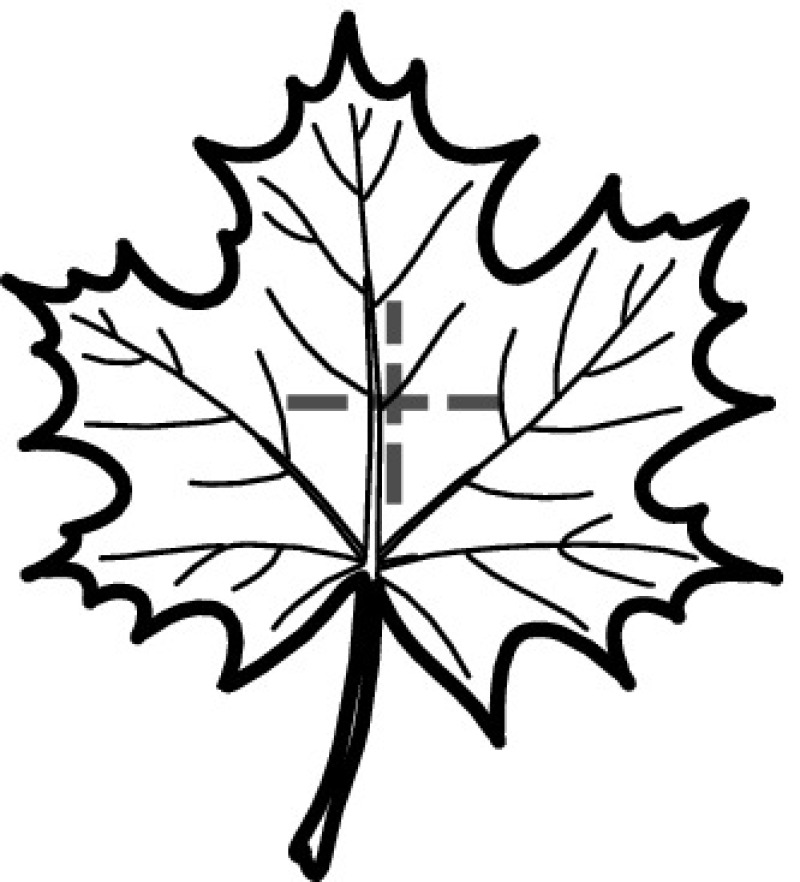		[82, 128]	2
*Aspect ratio* *AR* (aka slimness)	Ratio of *major axis length* to *minor axis length*—explains narrow or wide leaf or flower characteristics	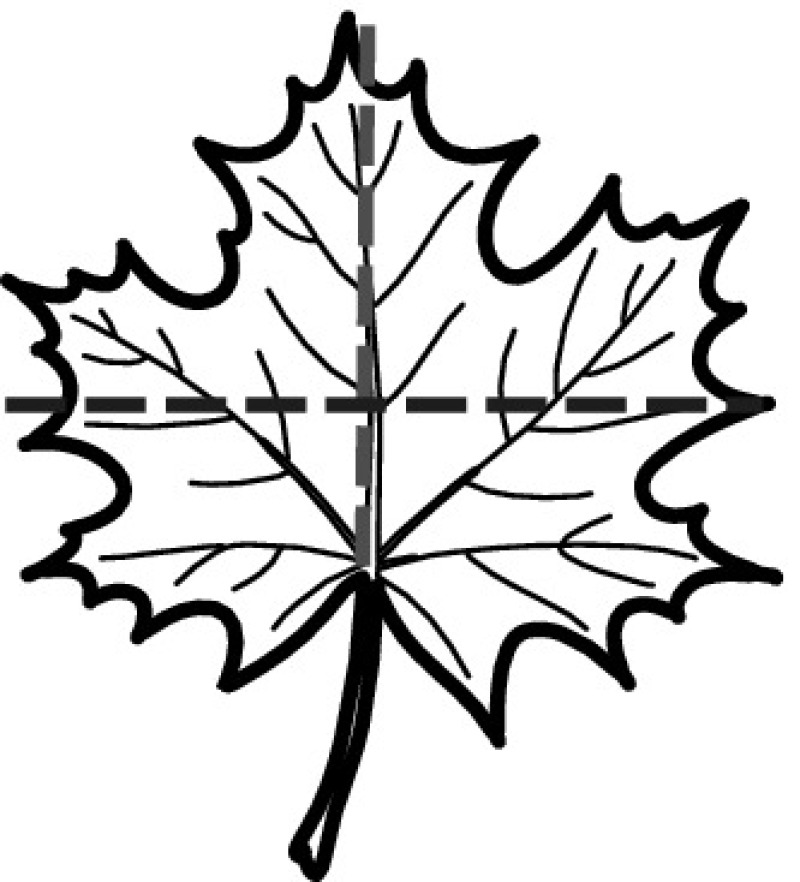	$AR=\frac{L}{W}$	[1, 3, 5, 15, 16, 24, 40, 43, 48, 72, 82, 89, 110, 116, 129, 137, 141, 144, 154]	19
*Roundness* *R* (aka form factor, circularity, isoperimetric factor)	Illustrates the difference between a organ and a circle	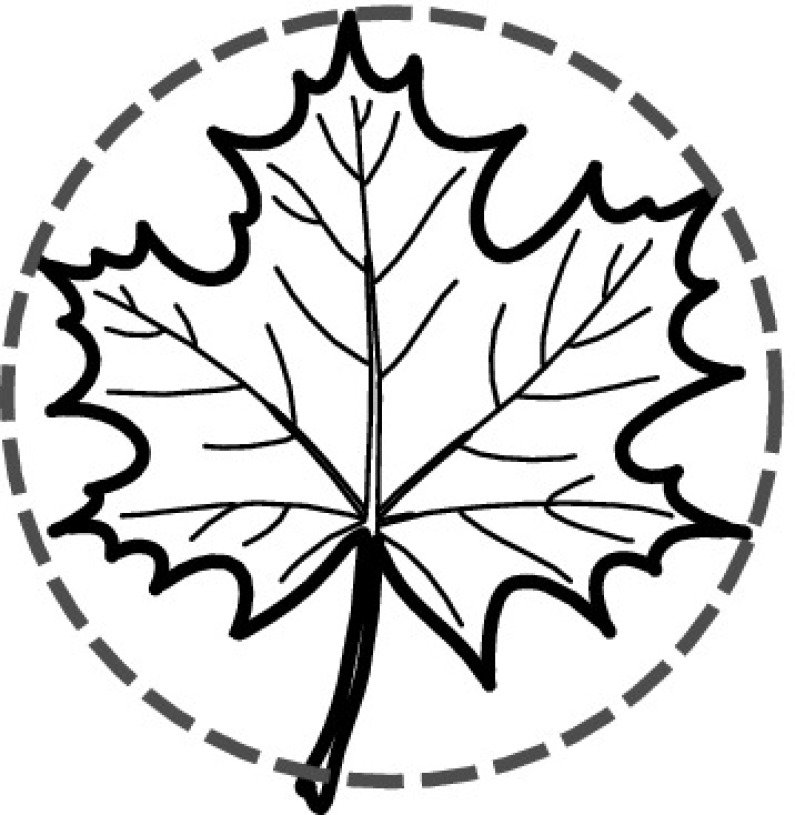	$R = \frac{4 \pi A}{P^{2}}$	[1, 3, 5, 16, 24, 40, 43, 48, 60, 72, 89, 110, 116, 137, 144, 146]	16
*Compactness* (aka perimeter ratio of area)	Ratio of the *perimeter* over the object’s *area*; provides information about the general complexity and the form factor, it is closely related to roundness	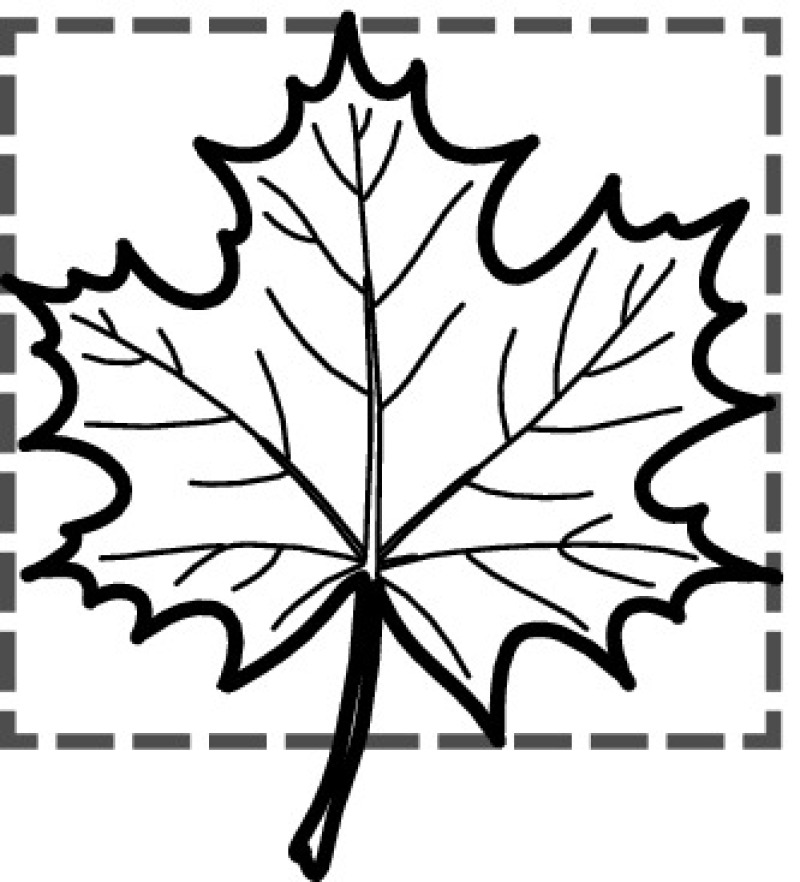	$C= \frac{P^2}{A} ; C= \frac{P}{\sqrt{A}}$	[73, 82, 148]	3
*Rectangularity* *N* (aka extent)	Represents how rectangle a shape is, i.e., how much it fills its minimum bounding rectangle	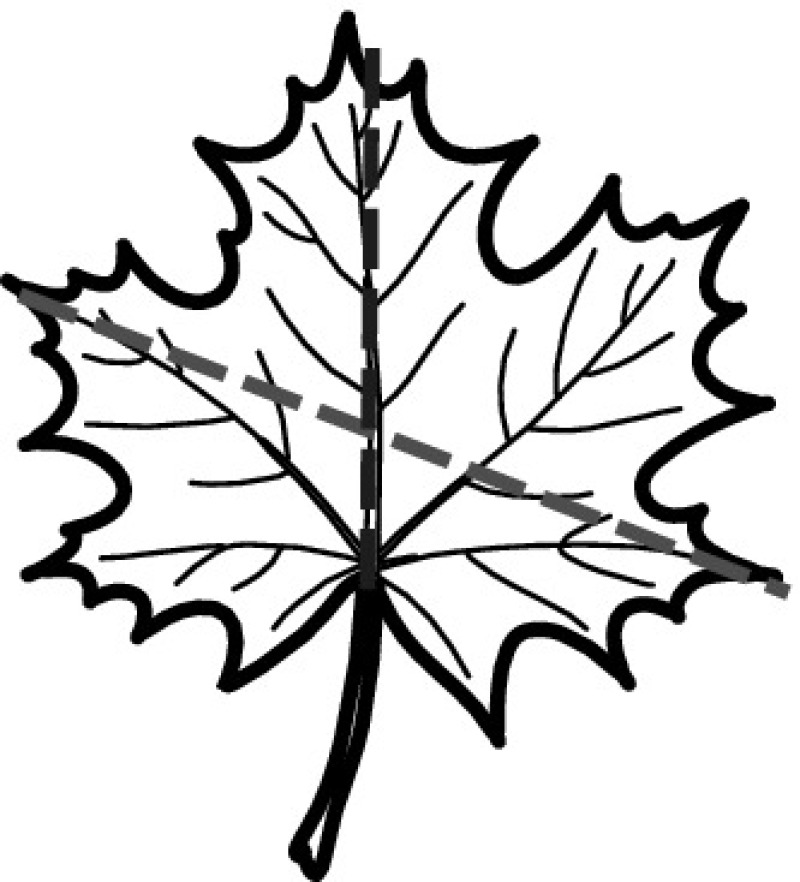	$N= \frac{A}{LW}$	[5, 16, 24, 40, 48, 58, 89, 144, 146]	10
*Eccentricity* *E*	Ratio of the distance between the foci of the ellipse (*f*) and its major axis length (*a*); computes to 0 for a round object and to 1 for a line		$E = \frac{f}{a}$	[1, 40, 43, 58, 110]	5
*Narrow factor* *NF*	Ratio of the *diameter* over the *Major axis length*	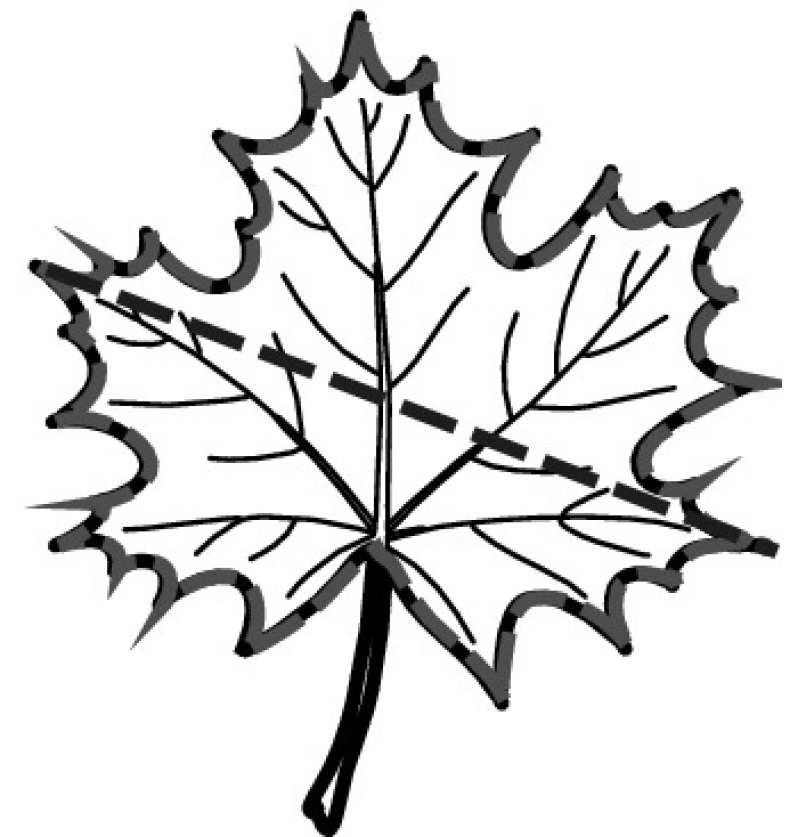	$NF= \frac{D}{L}$	[5, 16, 89, 137, 144]	5
*Perimeter ratio of diameter* $P_D$	Ratio of the *perimeter* to the *diameter*	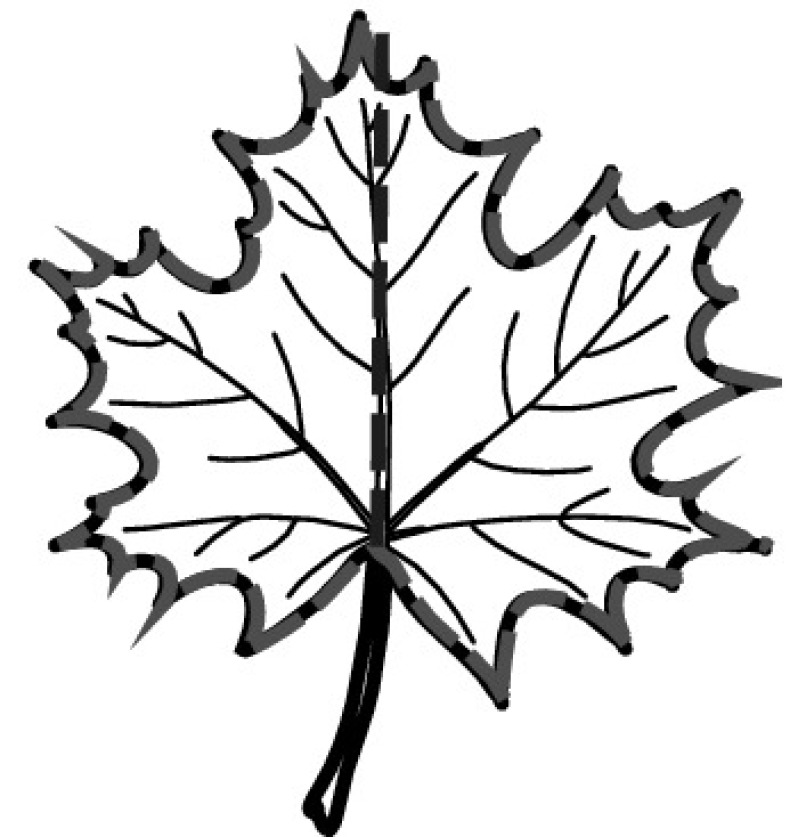	$P_D = \frac{P}{D}$	[5, 89, 144]	3
*Perimeter ratio of Major axis length* $P_L$	Ratio of the *perimeter* to the *major axis length*	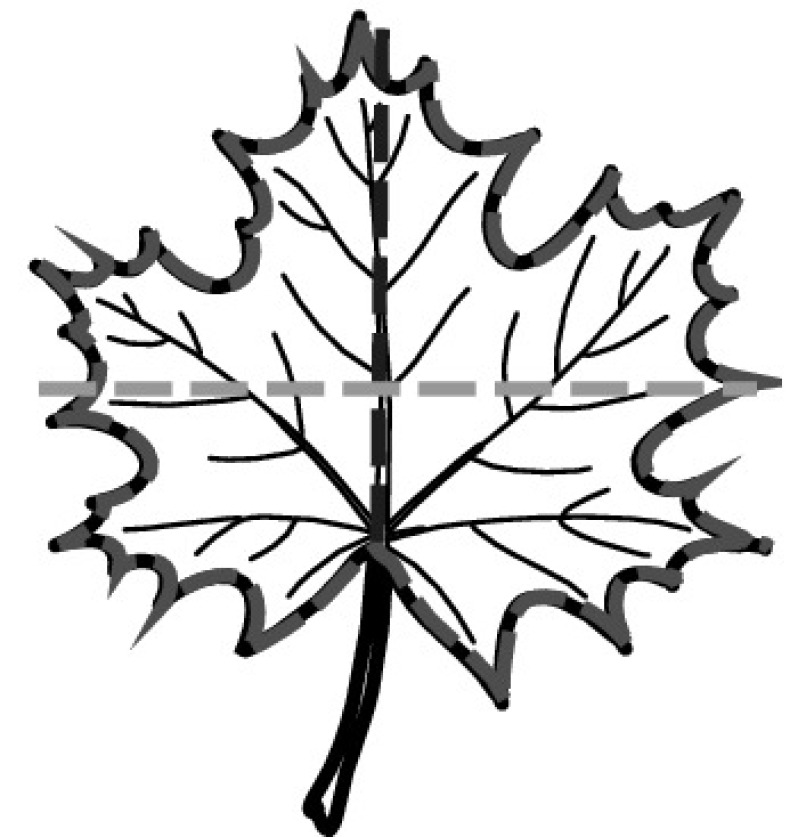	$P_L= \frac{P}{L}$	[16, 24, 89]	3
*Perimeter ratio of Major axis length and Minor axis length* $P_{LW}$	Ratio of object *perimeter* over the sum of the *major axis length* and the *minor axis length*	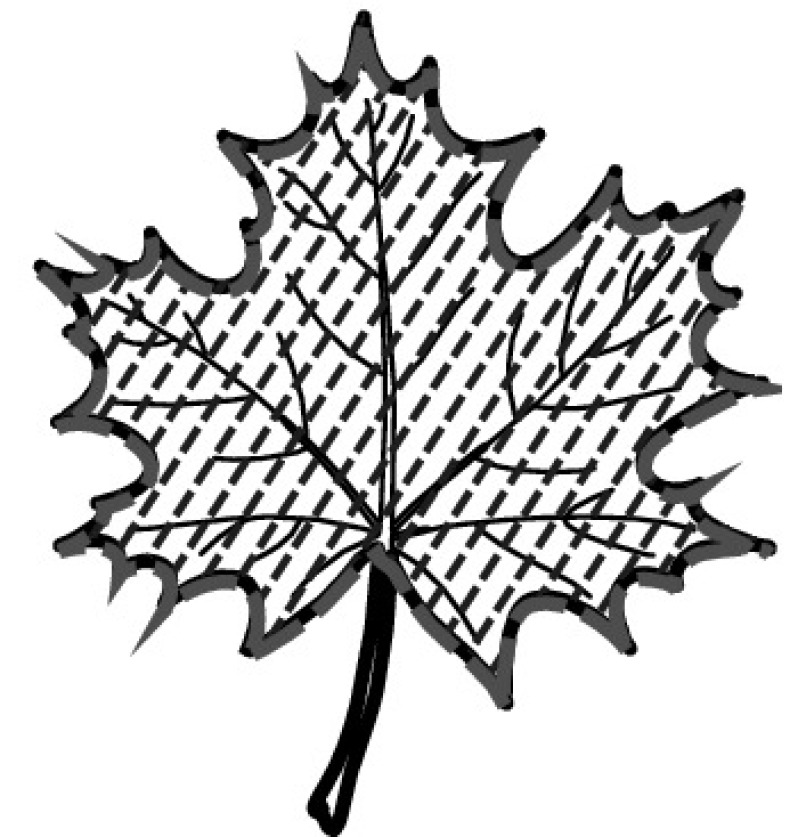	$P_{LW}= \frac{P}{(L +W)}$	[5, 16, 24, 89, 144]	5
*Convex hull* *CH* (aka Convex area)	The convex hull of a region is the smallest region that satisfies two conditions: (1) it is convex, and (2) it contains the organ’s region	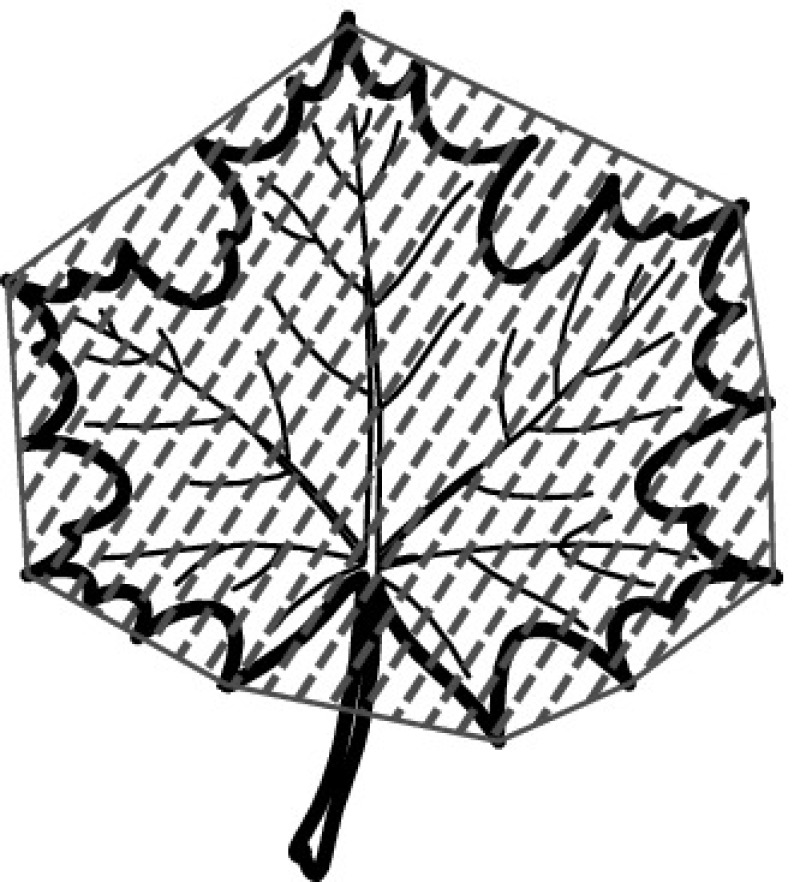		[1, 40, 58, 137]	4
*Perimeter convexity* $P_C$	Ratio of the *convex perimeter* $P_{CH}$ to the *perimeter* *P* of the organ	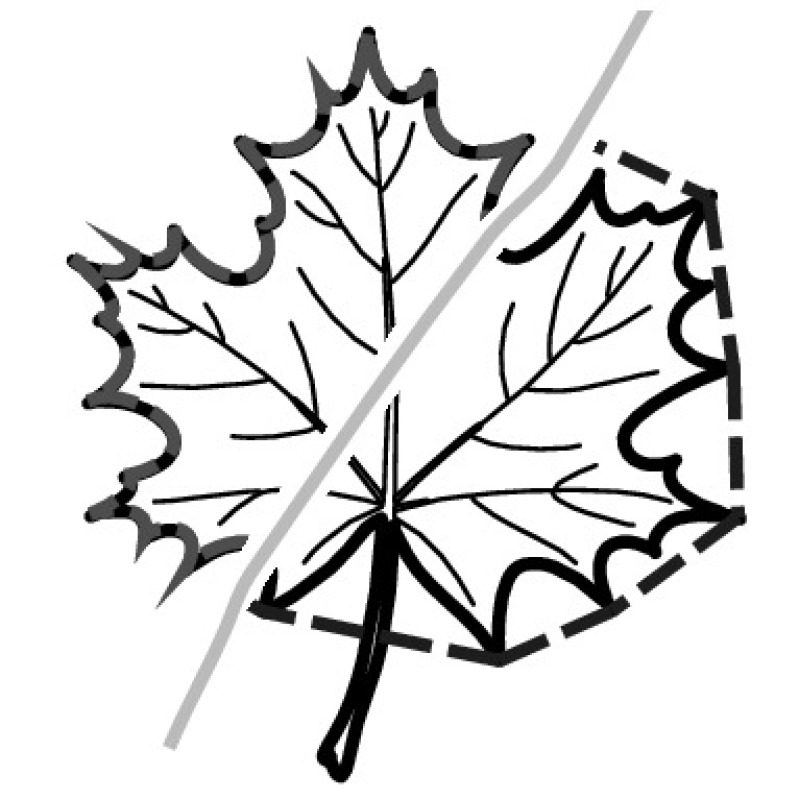	$P_C = \frac{P _{CH}}{P}$	[40, 73, 146, 148]	4
*Area convexity* $A_{C1}$ (aka Entirety)	Normalized difference of the *convex hull* area and the organ’s *area*	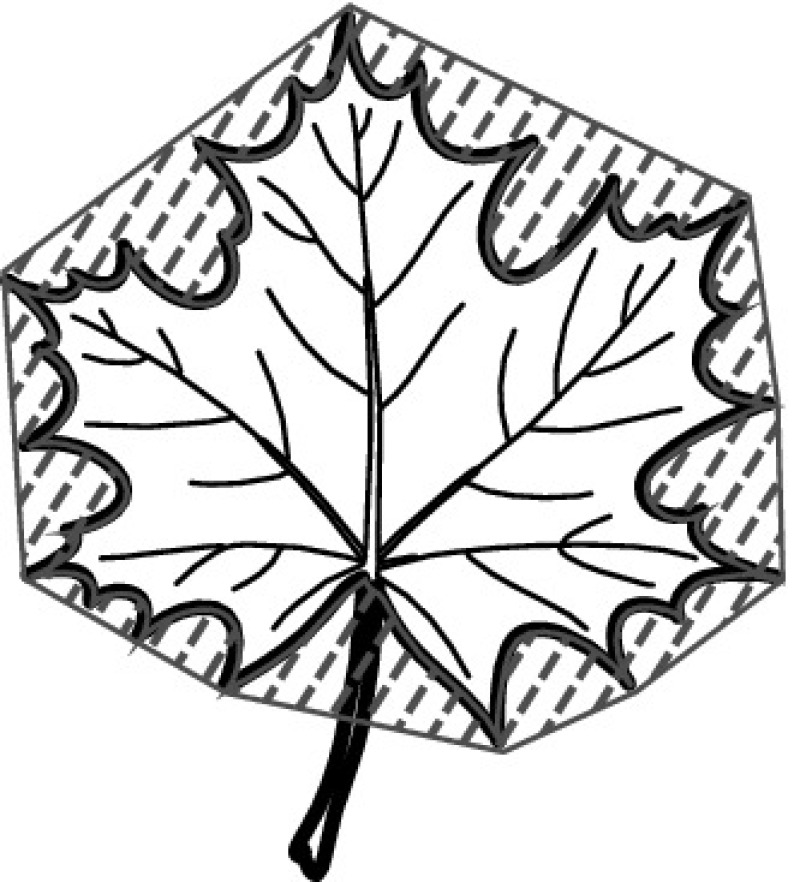	$A_{C1} = \frac{(CH - A)}{ A}$	[148]	1
*Area ratio of convexity* $A_{C2}$ (aka Solidity)	Ratio between organ’s *area* and area of the organ’s *convex hull*	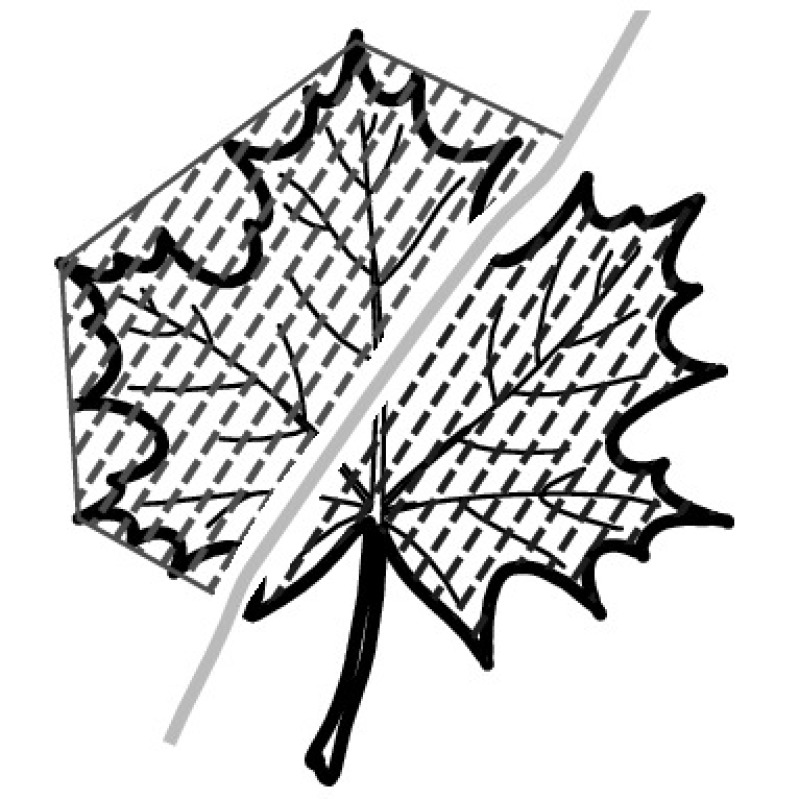	$A_{C2}= \frac{A}{CH}$	[40, 43, 73, 110, 137, 146]	6
*Sphericity* *S* (aka Dispersion)	Ratio of the radius of the inside circle of the bounding box ($r_i$) and the radius of the outside circle of the bounding box ($r_c$)	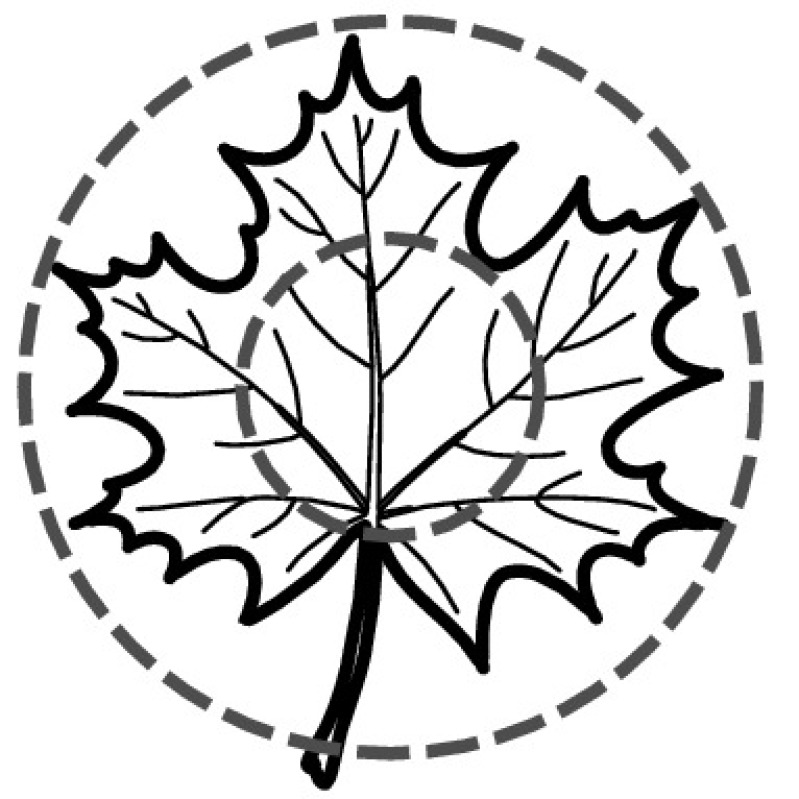	$S=\frac{r_i}{r_c}$	[40, 72, 137, 146]	4
*Equivalent diameter* $D_E$	Diameter of a circle with the same area as the organ’s *area*	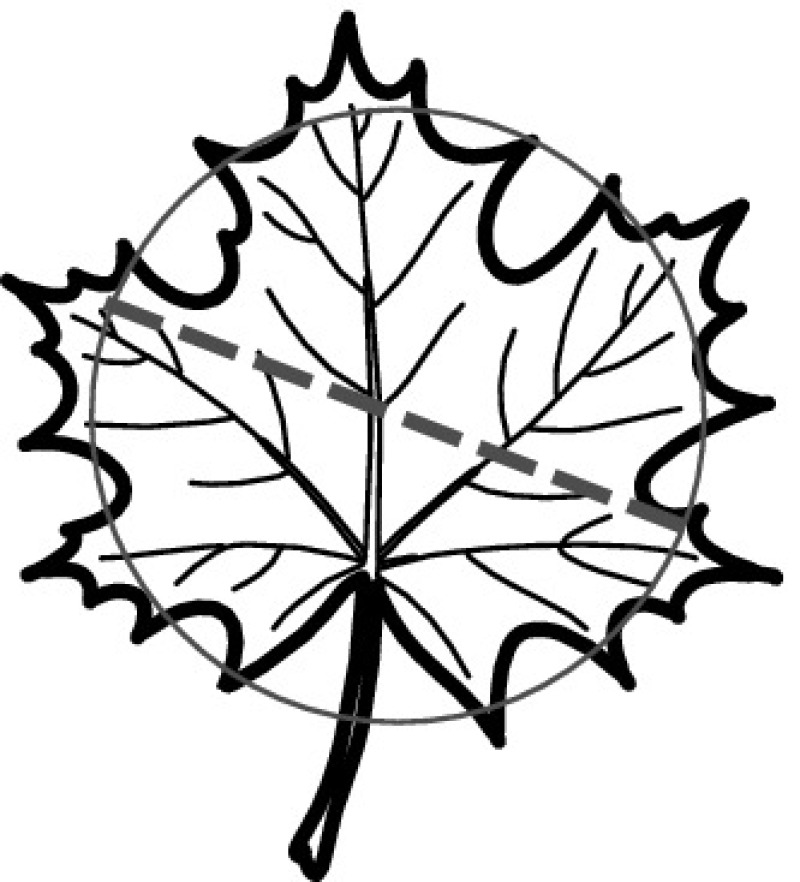	$D_E=\sqrt{ \frac{4*A}{\pi }}$	[58]	1
*Ellipse variance* *EA*	Represents the mapping error of a shape to fit an ellipse with same covariance matrix as the shape	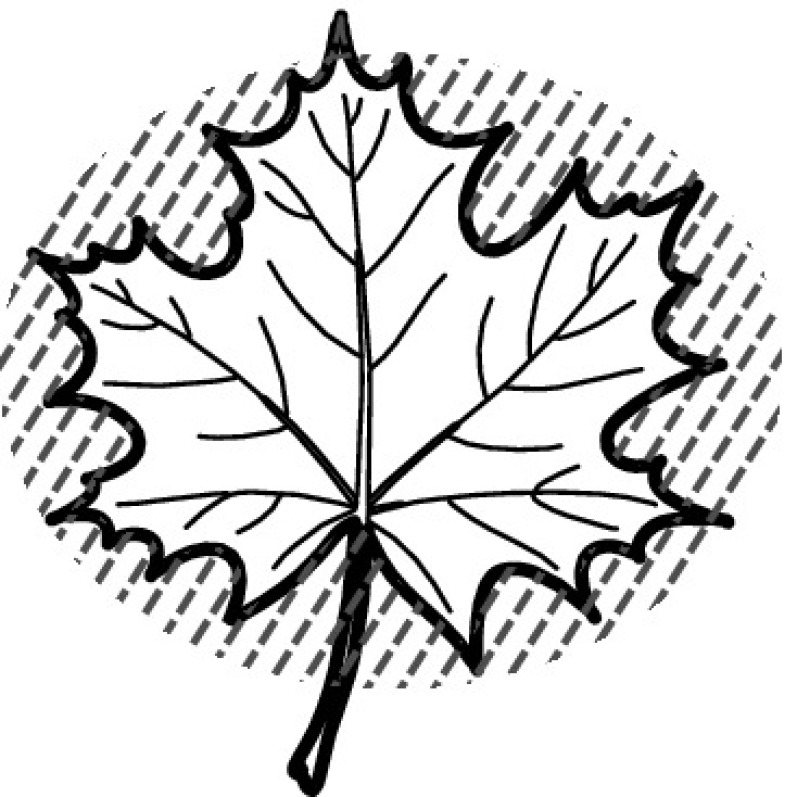		[141, 146]	2
*Smooth factor*	Ratio between organ’s *area* smoothed by a 5x5 rectangular averaging filter and one smoothed by a 2x2 rectangular averaging filter	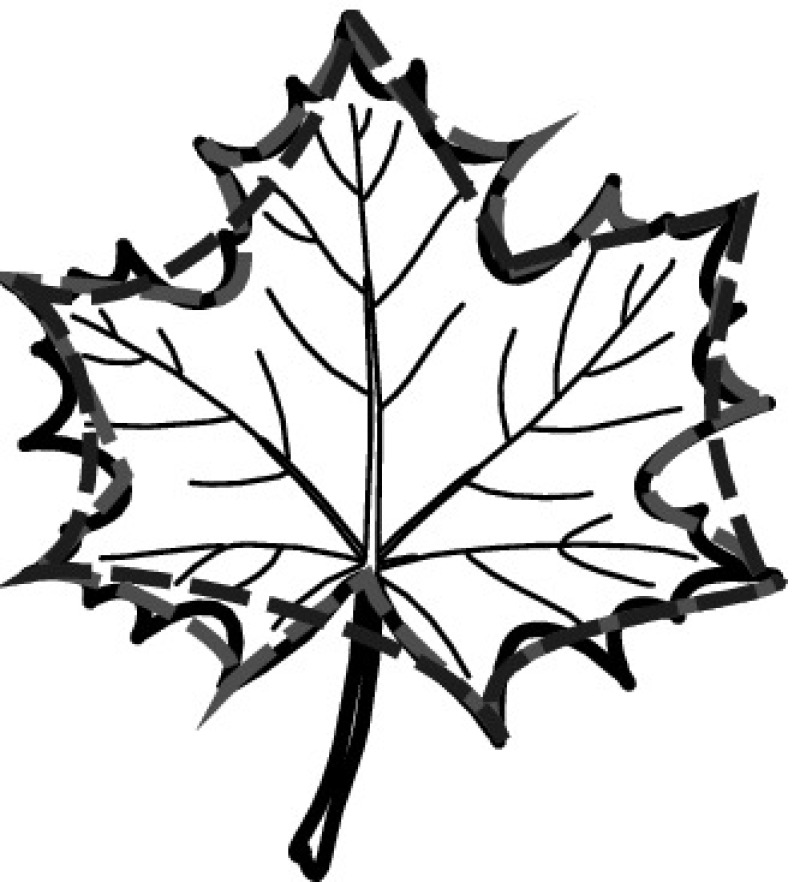		[5, 144]	2
*Leaf width factor LWF* $_c$	The leaf is sliced, perpendicular to the major axis, into a number of vertical strips. Then for each strip (*c*), the ratio of width of each strip ($W_c$) and the length of the entire leaf (*L*) is calculated	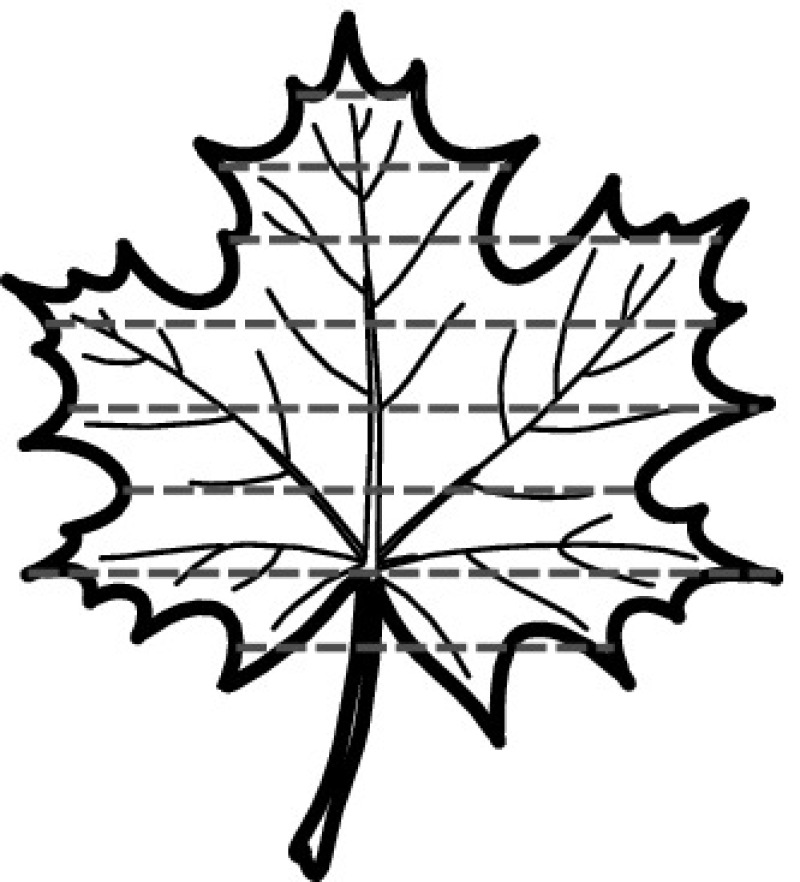	$LWF_c=\frac{W_c}{ L}$	[58]	1
*Area width factor AWF* $_c$	The leaf is sliced, perpendicular to the major axis, into a number of vertical strips. Then for each strip (*c*), the ratio of the area of each strip ($A_c$) and the area of the entire leaf (*A*) is calculated	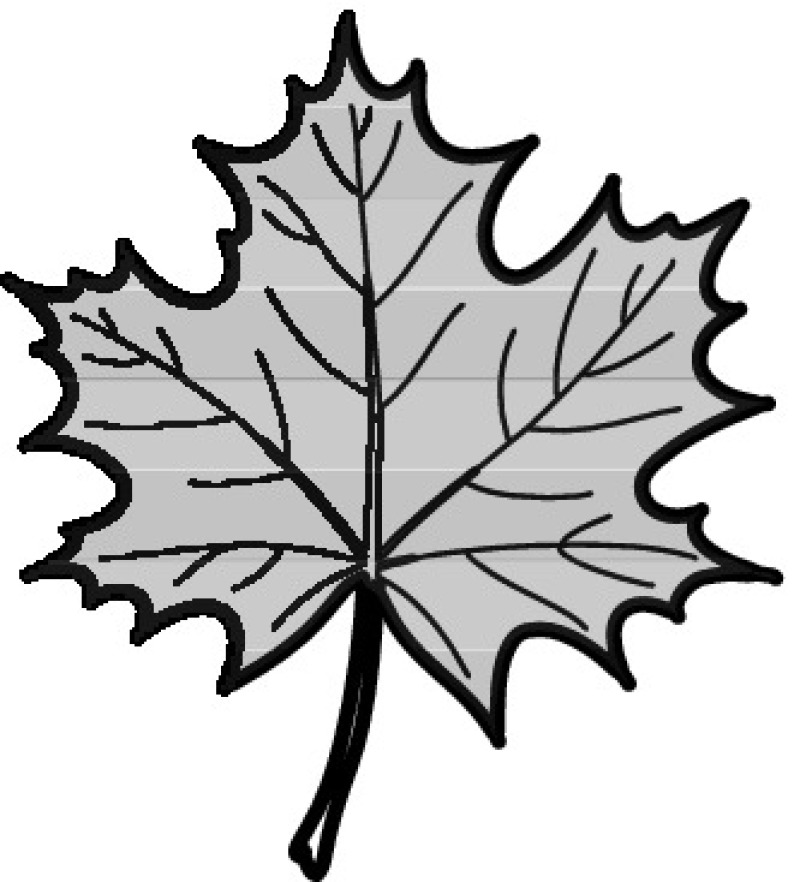	$AWF_c=\frac{A_c}{ A}$	[148]	1
*Porosity poro*	Portion of cracks in leaf image; $A_d$ is the detected area counting the holes in the object		$Poro={\frac{(A-A_d)}{ A}} *100\%$	[116]	1

However, while there typically exists high morphological variation across different species’ leaves, there is also often considerable variance among leaves of the same species. Studies’ results show that SMSD are too much simplified to discriminate leaves beyond those with large differences sufficiently. Therefore, they are usually combined with other descriptors, e.g., more complex shape analysis [1, 15, 40, 72, 73, 106, 110, 137, 146], leaf texture analysis [154], vein analysis [5, 144], color analysis [16, 116], or all of them together [43, 48]. SMSD are usually employed for high-level discrimination reducing the search space to a smaller set of species without losing relevant information and allowing to perform computationally more expensive operations at a later stage on a smaller search space [15].

Similarly, SMSD play an important role for **flower analysis**. Tan et al. [129] propose four flower shape descriptors, namely, area, perimeter of the flower, roundness of the flower, and aspect ratio. A simple scaling and normalization procedure has been employed to make the descriptors invariant to varying capture situations. The roundness measure and aspect ratio in combination with more complex shape analysis descriptors are used by [3] for analyzing flower shape.

In conclusion, the risk of SMSD is that any attempt to describe the shape of a leaf using only 5–10 descriptors may oversimplify matters to the extent that meaningful analysis becomes impossible, even if they seem sufficient to classify a small set of test images. Furthermore, many single-value descriptors are highly correlated with each other, making the task of choosing sufficiently independent features to distinguish categories of interest especially difficult [33].

#### Region-Based Shape Descriptors

Region-based techniques take all the pixels within a shape region into account to obtain the shape representation, rather than only using boundary information as the contour-based methods do. In this section, we discuss the most popular region-based descriptors for plant species identification: image moments and local feature techniques.

*Image moments.* Image moments are a widely applied category of descriptors in object classification. Image moments are statistical descriptors of a shape that are invariant to translation, rotation, and scale. Hu [61] proposes seven image moments, typically called *geometric moments* or *Hu moments* that attracted wide attention in computer vision research. Geometric moments are computationally simple, but highly sensitive to noise. Among our primary studies, geometric moments have been used for leaf analysis [22, 23, 40, 65, 72, 73, 102, 110, 137, 138, 154] as well as for flower analysis [3, 29]. Geometric moments as a standalone feature are only studied by [102]. Most studies combine geometric moments with the previously discussed SMSD [3, 23, 40, 72, 73, 110, 137, 154]. Also the more evolved *Zernike moment invariant (ZMI)* and *Legendre moment invariant (LMI)*, based on an orthogonal polynomial basis, have been studied for leaf analysis [72, 138, 159]. These moments are also invariant to arbitrary rotation of the object, but in contrast to geometric moments they are not sensitive to image noise. However, their computational complexity is very high. Kadir et al. [72] found ZMI not to yield better classification accuracy than geometric moments. Zulkifli et al. [159] compare three moment invariant techniques, ZMI, LMI, and moments of discrete orthogonal basis (aka *Tchebichef moment invariant (TMI)*) to determine the most effective technique in extracting features from leaf images. In result, the authors identified TMI as the most effective descriptor. Also [106] report that TMI achieved the best results compared with geometric moments and ZMI and were therefore used as supplementary features with lower weight in their classification approach.

*Local feature techniques.* In general, the concept of local features refers to the selection of scale-invariant keypoints (aka interest points) in an image and their extraction into local descriptors per keypoint. These keypoints can then be compared with those obtained from another image. A high degree of matching keypoints among two images indicates similarity among them. The seminal *Scale-invariant feature transform (SIFT)* approach has been proposed by [86]. SIFT combines a feature detector and an extractor. Features detected and extracted using the SIFT algorithm are invariant to image scale, rotation, and are partially robust to changing viewpoints and changes in illumination. The invariance and robustness of the features extracted using this algorithm makes it also suitable for object recognition rather than image comparison.

SIFT has been proposed and studied for leaf analysis by [26, 27, 59, 81]. A challenge that arises for object classification rather than image comparison is the creation of a codebook with trained generic keypoints. The classification framework by [26] combines SIFT with the Bag of Words (BoW) model. The BoW model is used to reduce the high dimensionality of the data space. Hsiao et al. [59] used SIFT in combination with sparse representation (aka sparse coding) and compared their results to the BoW approach. The authors argue that in contrast to the BoW approach, their sparse coding approach has a major advantage as no re-training of the classifiers for newly added leaf image classes is necessary. In [81], SIFT is used to detect corners for classification. Wang et al. [139] propose to improve leaf image classification by utilizing shape context (see below) and SIFT descriptors in combination so that both global and local properties of a shape can be taken into account. Similarly, [74] combines SIFT with global shape descriptors (high curvature points on the contour after chain coding). The author found the SIFT method by itself not successful at all and its accuracy significantly lower compared to the results obtained by combining it with global shape features. The original SIFT approach as well as all so far discussed SIFT approaches solely operate on grayscale images. A major challenge in leaf analysis using SIFT is often a lack of characteristic keypoints due to the leaves’ rather uniform texture. Using colored SIFT (CSIFT) can address this problem and will be discussed later in the section about color descriptors.

Another substantially studied local feature approach is the *histogram of oriented gradients (HOG)* descriptor [41, 111, 145, 155]. The HOG descriptor, introduced by [86] is similar to SIFT, except that it uses an overlapping local contrast normalization across neighboring cells grouped into a block. Since HOG computes histograms of all image cells and there are even overlap cells between neighbor blocks, it contains much redundant information making dimensionality reduction inevitably for further extraction of discriminant features. Therefore, the main focus of studies using HOG lies on dimensionality reduction methods. Pham et al. [111], Xiao et al. [145] study the maximum margin criterion (MMC), [41] studies principle component analysis (PCA) with linear discriminant analysis (LDA), and [155] introduce attribute-reduction based on neighborhood rough sets. Pham et al. [111] compared HOG features with Hu moments and the obtained results show that HOG is more robust than Hu moments for species classification. Xiao et al. [145] found that HOG-MMC achieves a better accuracy than the inner-distance shape context (IDSC) (will be introduced in the section about contour based shape descriptors), when leaf petiole were cut off before analysis. A disadvantage of the HOG descriptor is its sensitivity to the leaf petiole orientation while the petiole’s shape actually carrying species characteristics. To address this issue, a pre-processing step can normalize petiole orientation of all images in a dataset making them accessible to HOG [41, 155].

Nguyen et al. [103] studied *speeded up robust features (SURF)* for leaf classification, which was first introduced by [9]. The SURF algorithm follows the same principles and procedure as SIFT. However, details per step are different. The standard version of SURF is several times faster than SIFT and claimed by its authors to be more robust against image transformations than SIFT [9]. To reduce dimensionality of extracted features, [103] apply the previously mentioned BoW model and compared their results with those of [111]. SURF was found to provide better classification results than HOG [111].

Ren et al. [121] propose a method for building leaf image descriptors by using *multi-scale local binary patterns (LBP)*. Initially, a multi-scale pyramid is employed to improve leaf data utilization and each training image is divided into several overlapping blocks to extract LBP histograms in each scale. Then, the dimension of LBP features is reduced by a PCA. The authors found that the extracted multi-scale overlapped block LBP descriptor can provide a compact and discriminative leaf representation.

Local features have also been studied for **flower analysis**. Nilsback and Zisserman [104], Zawbaa et al. [149] used SIFT on a regular grid to describe shapes of flowers. Nilsback and Zisserman [105] proposed to sample HOG and SIFT on both, the foreground and its boundary. The authors found SIFT descriptors extracted from the foreground to perform best, followed by HOG, and finally SIFT extracted from the boundary of a flower shape. Combining foreground SIFT with boundary SIFT descriptors further improved the classification results.

Qi et al. [117] studied *dense SIFT (DSIFT)* features to describe flower shape. DSIFT is another SIFT-like feature descriptor. It densely selects points evenly in the image, on each pixel or on each n-pixels, rather than performing salient point detection, which make it strong in capturing all features in an image. But DSIFT is not scale-invariant, to make it adaptable to changes in scale, local features are sampled by different scale patches within an image [84]. Unlike the work of [104, 105], [117] take the full image as input instead of a segmented image, which means that extended background greenery may affect their classification performance to some extent. However, the results of [117] are comparable to the results of [104, 105]. When considering segmentation and complexity of descriptor as factors, the authors even claim that their method facilitates more accurate classification and performs more efficiently than the previous approaches.

#### Contour-Based Shape Descriptors

Contour-based descriptors solely consider the boundary of a shape and neglect the information contained in the shape interior. A contour-based descriptor for a shape is a sequence of values calculated at points taken around an object’s outline, beginning at some starting point and tracing the outline in either a clockwise or an anti-clockwise direction. In this section, we discuss popular contour-based descriptors namely shape signatures, shape context approaches, scale space, the Fourier descriptor, and fractal dimensions.

*Shape signatures*. Shape signatures are frequently used contour-based shape descriptors, which represent a shape by an one dimensional function derived from shape contour points. There exists a variety of shape signatures. We found the *centroid contour distance (CCD)* to be the most studied shape signature for leaf analysis [10, 28, 46, 130] and flower analysis [3, 57]. The CCD descriptor consists of a sequence of distances between the center of the shape and points on the contour of a shape. Other descriptors consist of a sequence of angles to represent the shape, e.g., the *centroid-angle (AC)* [10, 46] and the *tangential angle (AT)* [6]. A comparison between CCD and AC sequences performed by [46] demonstrated that CCD sequences are more informative than AC sequences. This observation is intuitive since the CCD distance includes both global information related to the leaf area and shape as well as local information related to contour details. Therefore, when combining CCD and AC, which is expected to further improve classification performance, the CCD should be emphasized by giving it a higher classification weight [46].

Mouine et al. [92] investigate two multi-scale triangular approaches for leaf shape description: the well-known *triangle area representation (TAR)* and the *triangle side length representation (TSL)*. The TAR descriptor is computed based on the area of triangles formed by points on the shape contour. TAR provides information about shape properties, such as the convexity or concavity at each contour point of the shape, and provides high discrimination capability. Although TAR is affine-invariant and robust to noise and deformation, it has a high computational cost since all the contour points are used. Moreover, TAR has two major limitations: (a) the area is not informative about the type of the considered triangle (isosceles, equilateral, etc.), which may be crucial for a local description of the contour. (b) The area is not accurate enough to represent the shape of a triangle [94]. The TSL descriptor is computed based on the side lengths rather than the area of a triangle. TSL is invariant under scale, translation, rotation, and reflection around contour points. Studies found TSL to provide yield higher classification accuracy than TAR [92, 94]. The authors argue that this result may be due to the fact that using side lengths to represent a triangle is more accurate than using its area. In addition to the two multi-scale triangular approaches, [94] also proposed two representations that they denote *triangle oriented angles (TOA)* and *triangle side lengths and angle representation (TSLA)*. TOA solely uses angle values to represent a triangle. Angle orientation provides information about local concavities and convexities. In fact, an obtuse angle means convex, an acute angle means concave. TOA is not invariant under reflection around the contour point: only similar triangles having equal angles will have equal TOA values. TSLA is a multi-scale triangular contour descriptor that describes the triangles by their lengths and angle. Like TSL, the TSLA descriptor is invariant under scale and reflection around the contour points. The authors found that the angular information provides a more precise description when being jointly used with triangle side lengths (i.e., TSL) [94].

A disadvantage of shape signatures for leaf and flower analysis is the high matching cost, which is too high for online retrieval. Furthermore, shape signatures are sensitive to noise and changes in the contour. Therefore, it is undesirable to directly describe a shape using a shape signature. On the other hand, further processing can increase its robustness and reduce the matching load. For example, a shape signature can be simplified by quantizing a contour into a contour histogram, which is then rotationally invariant [151]. For example, an *angle code histogram (ACH)* has been used instead of AC by [148]. However, the authors did not compare AC against ACH.

*Shape context approaches*. Beyond the CCD and AC descriptors discussed before, there are other alternative methods that intensively elaborate a shape’s contour to extract useful information. Belongie et al. [12] proposed a shape descriptor, called *shape context (SC)*, that represent log-polar histograms of contour distribution. A contour is resampled to a fixed number of points. In each of these points, a histogram is computed such that each bin counts the number of sampled contour points that fall into its space. In other words, each contour point is described by a histogram in the context of the entire shape. Descriptors computed in similar points on similar shapes will provide close histograms. However, articulation (e.g., relative pose of the petiole or the position of the blade) results in significant variation of the calculated SC. In order to obtain articulation invariance, [83] replaced the Euclidean distance and relative angles by inner-distances and inner-angles. The resulting 2D histogram, called *inner-distance shape context (IDSC)*, was reported to perform better than many other descriptors in leaf analysis [11]. It is robust to the orientation of the footstalk, but at the cost of being a shape descriptor that is extensive in size and expensive in computational cost. For example, [139] does not employ IDSC due to its expensive computational cost.

Hu et al. [62] propose a contour-based shape descriptor named *multi-scale distance matrix (MDM)* to capture the geometric structure of a shape, while being invariant to translation, rotation, scaling, and bilateral symmetry. The approach can use Euclidean distances as well as inner distances. MDM is considered a most effective method since it avoids the use of dynamic programming for building the point-wise correspondence. Compared to other contour-based approaches, such as SC and IDSC, MDM can achieve comparable recognition performance while being more computationally efficient [62]. Although MDM effectively describes the broad shape of a leaf, it fails in capturing details, such as leaf margin. Therefore, [73] proposed a method that combines contour (MDM), margin (average margin distance (AMD), margin statistics (MS)), SMSD and Hu moments and demonstrated higher classification accuracy than reached by using MDM and SMSD with Hu moments alone.

Zhao et al. [158] made two observations concerning shape context approaches. First, IDSC cannot model local details of leaf shapes sufficiently, because it is calculated based on all contour points in a hybrid way so that global information dominates the calculation. As a result, two different leaves with similar global shape but different local details tend to be misclassified as the same species. Second, the point matching framework of generic shape classification methods does not work well for compound leaves since their local details are hard to be matched in pairs. To solve this problem, [158] proposed an *independent-IDSC (I-IDSC)* feature. Instead of calculating global and local information in a hybrid way, I-IDSC calculates them independently so that different aspects of a leaf shape can be examined individually. The authors argue that compared to IDSC [11, 83] and MDM [62], the advantage of I-IDSC is threefold: (1) it discriminates leaves with similar overall shape but different margins and vice versa; (2) it accurately classifies both simple and compound leaves; and (3) it only keeps the most discriminative information and can thus be more efficiently computed [158].

Wang et al. [134, 135] developed a *multi scale-arch-height descriptor (MARCH)*, which is constructed based on the concave and convex measures of arches of various levels. This method extracts hierarchical arch height features at different chord spans from each contour point to provide a compact, multi-scale shape descriptor. The authors claim that MARCH has the following properties: invariant to image scale and rotation, compactness, low computational complexity, and coarse-to-fine representation structure. The performance of the proposed method has been evaluated and demonstrated to be superior to IDSC and TAR [134, 135].

*Scale space analysis*. A rich representation of a shape’s contour is the *curvature-scale space (CSS)*. It piles up curvature measures at each point of the contour over successive smoothing scales, summing up the information into a map where concavities and convexities clearly appear, as well as the relative scale up to which they persist [151]. Florindo et al. [45] propose an approach to leaf shape identification based on curvature complexity analysis (fractal dimension based on curvature). By using CSS, a curve describing the complexity of the shape can be computed and theoretically be used as descriptor. Studies found the technique to be superior to traditional shape analysis methods like FD, Zernike moments, and multi-scale fractal dimension [45]. However, while CSS is a powerful description it is too informative to be used as a descriptor. The implementation and matching of CSS is very complex. Curvature has also been used to detect dominant points (points of interest or characteristic points) on the contour, and provides a compact description of a contour by its curvature optima. Studies select this characteristic or the most prominent points based on the graph of curvature values of the contour as descriptor [15, 18]. Lavania and Matey [81] use *mean projection transform (MPT)* to extract corner candidates by selecting only candidates that have high curvature (contour-based edge detection). Kebapci et al. [74] extract high curvature points on the contour by analyzing direction changes in the chain code. They represent the contour as a chain code, which is a series of enumerated direction codes. These points (aka codes) are labeled as convex or concave depending on their position and direction (or curvature of the contour). Kumar et al. [76] suggest a leaf classification method using so-called *histograms of curvature over scale (HoCS)*. HoCS are built from CSS by creating histograms of curvature values over different scales. One limitation of the HoCS method is that it is not articulation-invariant, i.e., that a change caused by the articulation either between the blade and petiole of a simple leaf, or among the leaflets of a compound leaf can cause significant changes to the calculated HoCS feature. Therefore, it needs special treatment of leaf petioles and the authors suggest to detect and remove the petiole before classification. Chen et al. [28] used a simplified curvature of the leaf contour, called velocity. The results showed that the velocity algorithms were faster at finding contour shape characteristics and more reasonable in their characteristic matching than CSS. Laga et al. [77] study the performance of the *squared root velocity function (SRVF)* representation of closed planar curves for the analysis of leaf shapes and compared it to IDSC, SC, and MDM. SRVF significantly outperformed the previous shape-based techniques. Among the lower performing techniques in this study, SC and MDM performed equally, IDCS achieved the lowest performance.

*Fourier descriptors*. *Fourier descriptors (FD)* are a classical method for shape recognition and have grown into a general method to encode various shape signatures. By applying a Fourier transform, a leaf shape can be analyzed in the frequency domain, rather than the spatial domain as done with shape signatures. A set number of Fourier harmonics are calculated for the outline of an object, each consisting of only four coefficients. These Fourier descriptors capture global shape features in the low frequency terms (low number of harmonics) and finer features of the shape in the higher frequency terms (higher numbers of harmonics). The advantages of this method are that it is easy to implement and that it is based on the well-known theory of Fourier analysis [33]. FD can easily be normalized to represent shapes independently of their orientation, size, and location; thus easing comparison between shapes. However, a disadvantage of FDs is that they do not provide local shape information since this information is distributed across all coefficients after the transformation [151]. A number of studies focused on FD, e.g., [147] use FD computed on distances of contour points from the centroid, which is advantageous for smaller datasets. Kadir et al. [72] propose a descriptor based on *polar Fourier transform (PFT)* to extract the shape of leaves and compared it with SMSD, Hu, and Zernike moments. Among those methods, PFT achieved the most prospective classification result. Aakif and Khan [1], Yanikoglu et al. [148] used FD in combination with SMSD. The authors obtained more accurate classification results by using FD than with SMSD alone. However, they achieved the best result by combining all descriptors. Novotny and Suk [106] used FD in combination with TMI and major axis length. Several studies that propose novel methods for leaf shape analysis benchmark their descriptor against FD in order to prove effectiveness [45, 62, 134, 147, 158].

*Fractal dimension*. The *fractal dimension (FracDim)* of an object is a real number used to represent how completely a shape fills the dimensional space to which it belongs. The FracDim descriptor can provide a useful measure of a leaf shape’s complexity. In theory, measuring the fractal dimension of leaves or flowers can quantitatively describe and classify even morphologically complex plants. Only a few studies used FracDim for leaf analysis [14, 65, 67] and flower analysis [3]. Bruno et al. [14] compare box-counting and multi-scale Minkowski estimates of fractal dimension. Although the box-counting method provided satisfactory results, Minkowski’s multi-scale approach proved superior in terms of characterizing plant species. Given the wide variety of leaf and flower shapes, characterizing their shape by a single value descriptor of complexity likely discards useful information, suggesting that the FracDim descriptors may only be useful in combination with other descriptors. For example, [65] demonstrated that leaf analysis with FracDim descriptors are effective and yield higher classification rates than Hu moments. When combining both, even better results were achieved. One step further, [14, 65, 67] proposed methods for combining the FD descriptor of a leaf’s shape with a FracDim descriptor computed on the venation of the leaf to rise classification performance (further details in the section about vein feature).

#### Color

Color is an important feature of images. Color properties are defined within a particular color space. A number of color spaces have been applied across the primary studies, such as red-green-blue (RGB), hue-saturation-value (HSV), hue-saturation-intensity (HSI), hue-max-min-diff (HMMD), LUV (aka CIELUV), and more recently Lab (aka CIELAB). Once a color space is specified, color properties can be extracted from images or regions. A number of general color descriptors have been proposed in the field of image recognition, e.g., *color moments (CM), color histograms (CH), color coherence vector*, and *color correlogram* [153]. CM are a rather simple descriptor, the common moments being mean, standard deviation, skewness, and kurtosis. CM are used for characterizing planar color patterns, irrespective of viewpoint or illumination conditions and without the need for object contour detection. CM is known for its low dimension and low computational complexity, thus, making it convenient for real-time applications. CH describes the color distribution of an image. It quantizes a color space into different bins and counts the frequency of pixels belonging to each color bin. This descriptor is robust to translation and rotation. However, CH does not encode spatial information about the color distribution. Therefore, visually different images can have similar CH. In addition, a histogram is usually of high dimensionality [153]. A major challenge for color analysis is light variations due to different intensity and color of the light falling from different angles. These changes in illumination can cause shadowing effects and intensity changes. For species classification the most studied descriptors are CM [16, 43, 48, 87, 116, 148] and CH [3, 16, 29, 57, 74, 87, 104, 105, 112, 148]. An overview of all primary studies that analyze color is shown in Table 8.

*Leaf analysis.* Only 8 of 106 studies applying leaf analysis also study color descriptors. We always found color descriptors being jointly studied together with leaf shape descriptors. Kebapci et al. [74] use three different color spaces to produce CH and *color co-occurrence matrices (CCM)* for assessing the similarity between two images; namely RGB, normalized RGB (nRGB), and HSI, where nRGB-CH facilitated the best results. Yanikoglu et al. [148] studied the effectiveness of color descriptors, specifically the RGB histogram and CM. The authors found CM to provide the most accurate results. However, the authors also found that color information did not contribute to the classification accuracy when combined with shape and texture descriptors. Caglayan et al. [16] defined different sets of color features. The first set consisted of mean and standard deviation of intensity values of the red, the green, and the blue channel and an average of these channels. The second set consisted of CH in red, green, and blue channels. The authors found the first four CM to be an efficient and effective way for representing color distribution of leaf images [43, 48, 116, 148]. Che Hussin et al. [27] proposed a grid-based CM as descriptor. Each image is divided into a 3x3 grid, then each cell is described by mean, standard deviation, and the third root of the skewness. In contrast, [87] evaluated the first three central moments, which they found to be not discriminative according their experimental results.

*Flower analysis.* Color plays a more important role for flower analysis than for leaf analysis. We found that 9 out of 13 studies on flower analysis use color descriptors. However, using color information solely, without considering flower shape features, cannot classify flowers effectively [104, 105]. Flowers are often transparent to some degree, i.e., that the perceived color of a flower differs depending on whether the light comes from behind or in front of the flower. Since flower images are taken under different environmental conditions, the variation in illumination is greatly affecting analysis results [126]. To deal with this problem, [3, 30, 57, 60] converted their images from the RGB color space into the HSV space and discarded the illumination (V) component. Apriyanti et al. [3] studied discrimination power of features for flower images and identified the following relation from the highest to the lowest: CCD (shape), HSV color, and geometric moments (shape). Hsu et al. [60] found that color features have more discriminating ability than the center distance sequence and the roundness shape features. Qi et al. [117] study a method where they select local keypoints with *colored SIFT (CSIFT)*. CSIFT is a SIFT-like descriptor that builds on a color invariants. It employs the same strategy as SIFT for building descriptors. The local gradient-orientation histograms for the same-scale neighboring pixels of a keypoint are used as descriptor. All orientations are assigned relative to a dominant orientation of the keypoint. Thus, the built descriptor is invariant to the global object orientation and is stable to occlusion, partial appearance, and cluttered surroundings due to the local description of keypoints. As CSIFT uses color invariants for building the descriptor, it is robust to photometric changes [2]. Qi et al. [117] even found the performance of CSIFT to be superior over SIFT.

**Table 8:** Studies analyzing the color of organs in combination with other features

Organ	Feature	Color descriptor	Studies
Leaf	Shape, **color**	CM, CH	[16, 87]
		CM	[27, 116]
	Shape, **color**, texture	CH, CCM	[74]
		CM, CH	[148]
	Shape, **color**, texture, vein	CM	[43, 48]
Flower	**Color**, shape	CH	[3, 30, 57, 60]
		CSIFT	[117]
	Shape, **color**, texture	CH	[29, 68, 104, 105, 112]
Full plant	Shape, **color**, texture	CH	[68]

#### Texture

Texture is the term used to characterize the surface of a given object or phenomenon and is undoubtedly a main feature used in computer vision and pattern recognition [142, 153]. Generally, texture is associated to the feel of different materials to human touch. Texture image analysis is based on visual interpretation of this feeling. Compared to color, which is usually a pixel property, texture can only be assessed for a group of pixels [153]. Grayscale texture analysis methods are generally grouped into four categories: signal processing methods based on a spectral transform, such as, Fourier descriptors (FD) and Gabor filters (GF); statistical methods that explore the spatial distribution of pixels, e.g., co-occurrence matrices; structural methods that represent texture by primitives and rules; and model-based methods based on fractal and stochastic models. However, some recently proposed methods cannot be classified into these four categories. For instance, methods based on deterministic walks, fractal dimension, complex networks, and gravitational models [37]. An overview of all primary studies that analyze texture is shown in Table 9.

*Leaf analysis.* For leaf analysis, twelve studies analyzed texture solely and another twelve studies combined texture with other features, i.e., shape, color, and vein. The most frequently studied texture descriptors for leaf analysis are *Gabor filter (GF)* [17, 23, 32, 74, 132, 150], *fractal dimensions (FracDim)* [7, 8, 36, 37], and *gray level co-occurrence matrix (GLCM)* [23, 32, 43, 48].

GF are a group of wavelets, with each wavelet capturing energy at a specific frequency and in a specific direction. Expanding a signal provides a localized frequency description, thereby capturing the local features and energy of the signal. Texture features can then be extracted from this group of energy distributions. GF has been widely adopted to extract texture features from images and has been demonstrated to be very efficient in doing so [152]. Casanova et al. [17] applied GF on sample windows of leaf lamina without main venation and leaf margins. They observed a higher performance of GF than other traditional texture analysis methods such as FD and GLCM. Chaki et al. [23], Cope et al. [32] combined banks of GF and computed a series of GLCM based on individual results. The authors found the performance of their approach to be superior to standalone GF and GLCM. Yanikoglu et al. [148] used GF and HOG for texture analysis and found GF to have a higher discriminatory power. Venkatesh and Raghavendra [132] proposed a new feature extraction scheme termed *local Gabor phase quantization (LGPQ)*, which can be viewed as the combination of GF with a local phase quantization scheme. In a comparative analysis the proposed method outperformed GF as well as the *local binary pattern (LBP)* descriptor.

Natural textures like leaf surfaces do not show detectable quasi-periodic structures but rather have random persistent patterns [63]. Therefore, several authors claim fractal theory to be better suited than statistical, spectral, and structural approaches for describing these natural textures. Authors found the volumetric *fractal dimension (FracDim)* to be very discriminative for the classification of leaf textures [8, 122]. Backes and Bruno [7] applied multi-scale volumetric FracDim for leaf texture analysis. de M Sa Junior et al. [36, 37] propose a method combining gravitational models with FracDim and lacunarity (counterpart to the FracDim that describes the texture of a fractal) and found it to outperform FD, GLCM, and GF.

Surface gradients and venation have also been exploited using the *edge orientation histogram descriptor (EOH)* [10, 10, 91, 148]. Here the orientations of edge gradients are used to analyze the macro-texture of the leaf. In order to exploit the venation structure, [25] propose the *EAGLE descriptor* for characterizing leaf edge patterns within a spatial context. EAGLE exploits the vascular structure of a leaf within a spatial context, where the edge patterns among neighboring regions characterize the overall venation structure and are represented in a histogram of angular relationships. In combination with *SURF*, the studied descriptors are able to characterize both local gradient and venation patterns formed by surrounding edges.

Elhariri et al. [43] studied first and second order statistical properties of texture. First order statistical properties are: average intensity, average contrast, smoothness, intensity histogram’s skewness, uniformity, and entropy of *grayscale intensity histograms (GIH)*. Second order statistics (aka statistics from GLCM) are well known for texture analysis and are defined over an image to be the distribution of co-occurring values at a given offset [55]. The authors found that the use of first and second order statistical properties of texture improved classification accuracy compared to using first order statistical properties of texture alone. Ghasab et al. [48] derive statistics from GLCM, named contrast, correlation, energy, homogeneity, and entropy and combined them with shape, color, and vein features. Wang et al. [136] used *dual-scale decomposition and local binary descriptors (DS-LBP)*. DS-LBP descriptors effectively combine texture and contour of a leaf and are invariant to translation and rotation.

*Flower analysis.* Texture analysis also plays an important role for flower analysis. Five of the 13 studies analyze the texture of flowers, whereby texture is always analyzed in combination with shape or color. Nilsback and Zisserman [104, 105] describe the texture of flowers by convolving the images with a *Leung-Malik (MR) filter bank*. The filter bank contains filters with multiple orientations. Zawbaa et al. [149] propose the *segmentation-based fractal texture analysis (SFTA)* to analyze the texture of flowers. SFTA breaks the input image into a set of binary images from which region boundaries’ FracDim are calculated and segmented texture patterns are extracted.

**Table 9:** Studies analyzing the texture of organs solely or in combination with other features

Organ	Feature	Texture descriptor	Studies
Leaf	**Texture**	GF	[17, 150]
		GF, GLCM	[32]
		LGPQ	[131]
		FracDim	[7, 8, 36, 36, 122]
		CT	[115]
		EAGLE, SURF	[25]
		[No information]	[118]
	Shape, **texture**	DWT	[154]
		EOH	[10]
		Fourier, EOH	[68, 91]
		RSC	[114]
		DS-LBP	[136]
		EnS	[140]
		GF, GLCM	[23]
		Gradient histogram	[143]
	Shape, color, **texture**	GF	[74]
		EOH, GF	[148]
	Shape, color, **texture**, vein	GIH, GLCM	[43]
		GLCM	[48]
Flower	Shape, **texture**	SFTA	[149]
	Shape, color, **texture**	Statistical attributes (mean, sd)	[29]
		EOH	[112]
		Fourier, EOH	[68]
		Leung-Malik filter bank	[104, 105]
Fruit, bark	Shape, **texture**	Fourier, EOH	[68]
Full plant	Shape, color, **texture**	Fourier, EOH	[68]

Abbreviations not explained in the text—*CT* curvelet transform, *DWT* discrete wavelet transform, *EnS* entropy sequence, *Fourier* Fourier histogram, *RSC* relative sub-image coefficients

#### Leaf-Specific Features

*Leaf venation.* Veins provide leaves with structure and a transport mechanism for water, minerals, sugars, and other substances. Leaf veins can be, e.g., parallel, palmate, or pinnate. The vein structure of a leaf is unique to a species. Due to a high contrast compared to the rest of the leaf blade, veins are often clearly visible. Analyzing leaf vein structure, also referred to as leaf venation, has been proposed in 16 studies (see Table 10).

Only four studies solely analyzed venation as a feature discarding any other leaf features, like, shape, size, color, and texture [53, 78–80]. Larese et al. [78–80] introduced a framework for identifying three legumes species on the basis of leaf vein features. The authors computed 52 measures per leaf patch (e.g., the total number of edges, the total number of nodes, the total network length, median/min/max vein length, median/min/max vein width). Larese et al. [80] defines and discusses each measure. The author [80] performed an experiment using images that were cleared using a chemical process (enhancing high contrast leaf veins and higher orders of visible veins), which increased their accuracy from 84.1 to 88.4% compared to uncleared images at the expense of time and cost for clearing. Gu et al. [53] processed the vein structure using a series of wavelet transforms and Gaussian interpolation to extract a leaf skeleton that was then used to calculate a number of run-length features. A run-length feature is a set of consecutive pixels with the same gray level, collinear in a given direction, and constituting a gray level run. The run length is the number of pixels in the run and the run length value is the number of times such a run occurs in an image. The authors obtained a classification accuracy of 91.2% on a 20 species dataset.

Twelve studies analyzed venation in combination with the shape of leaves [4, 5, 14, 65, 67, 101, 107, 108, 139, 144] and two studies analyzed venation in combination with shape, texture, and color [43, 48]. Nam et al. [101], Park et al. [107, 108] extract structure features in order to categorize venation patterns. Park et al. [107, 108] propose a leaf image retrieval scheme, which analyzes the venation of a leaf sketch drawn by the user. Using the curvature scale scope corner detection method on the venation drawing they categorize the density of feature points (end points and branch points) by using non-parametric estimation density. By extracting and representing these venation types, they could improve the classification accuracy from 25 to 50%. Nam et al. [101] performed classification on graph representations of veins and combined it with modified minimum perimeter polygons as shape descriptor. The authors found their method to yield better results than CSS, CCD, and FD. Four groups of researchers [5, 43, 48, 144] studied the ratio of vein-area (number of pixels that represent venation) and leaf-area ($A_{vein}/ A_{leaf}$) after morphological opening. Elhariri et al. [43], Ghasab et al. [48] found that using a combination of all features (vein, shape, color, and texture) yielded the highest classification accuracy. Wang et al. [139] used SC and SIFT extracted from contour and vein sample points. They noticed that vein patterns are not always helpful for SC based classification. Since in their experiments, vein extraction based on simple Canny edge detection generated noisy outputs utilizing the resulting vein patterns in shape context led to unstable classification performance. The authors claim that this problem can be remedied with advanced vein extraction algorithms [139]. Bruno et al. [14], Ji-Xiang et al. [65] and Jobin et al. [67] studied FracDim extracted from the venation and the outline of leafs and obtained promising results. Bruno et al. [14] argues that the segmentation of a leaf venation system is a complex task, mainly due to low contrast between the venation and the rest of the leaf blade structure. The authors propose a methodology divided into two stages: (i) chemical leaf clarification, and (ii) segmentation by computer vision techniques. Initially, the fresh leaf collected in the herbarium, underwent a chemical process of clarification. The purpose was removing the genuine leaf pigmentation. Then, the fresh leaves were digitalized by a scanner. Ji-Xiang et al. [65], Jobin et al. [67] did not use any chemical or biological procedure to physically enhance the leaf veins. They obtained a classification accuracy of 87% on a 30 species dataset and 84% on a 50 species dataset, respectively.

*Leaf margin.* All leaves exhibit margins (leaf blade edges) that are either serrated or unserrated. Serrated leaves have teeth, while unserrated leaves have no teeth and are described as being smooth. These margin features are very useful for botanists when describing leaves, with typical descriptions including details such as the tooth spacing, number per centimeter, and qualitative descriptions of their flanks (e.g., convex or concave). Leaf margin has seen little use in automated species identification with 8 out of 106 studies focusing on it (see Table 10). Studies usually combine margin analysis with shape analyses [18, 20, 21, 73, 85, 93]. Two studies used margin as sole feature for analysis [31, 66].

Jin et al. [66] propose a method based on morphological measurements of leaf tooth, discarding leaf shape, venation, and texture. The studied morphological measurements are the total number of teeth, the ratio between the number of teeth and the length of the leaf margin expressed in pixels, leaf-sharpness, and leaf-obliqueness. Leaf-sharpness, is measured per tooth as an acute triangle obtained by connecting the top edge and two bottom edges of the leaf tooth. Thus, for a leaf image, many triangles corresponding to leaf teeth are obtained. In their method, the acute angle for each leaf tooth is exploited as a measure for plant identification. The proposed method achieves an average classification rate of around 76% for the eight studied species. Cope and Remagnino [31] extracts a margin signature based on the leaf’s insertion point and apex. A classification accuracy of 91% was achieved on a larger dataset containing 100 species. The authors argue that accurate identification of insertion point and apex may also be useful when considering other leaf features, e.g., venation. Two shape context based descriptors have been presented and combined for plant species identification by [93]. The first one gives a description of the leaf margin. The second one computes the spatial relations between the salient points and the leaf contour points. Results show that a combination of margin and shape improved classification performance in contrast to using them as separate features. Kalyoncu and Toygar [73] use margin statistics over margin peaks, i.e., average peak height, peak height variance, average peak distance, and peak distance variance, to describe leave margins and combined it with simple shape descriptors, i.e., Hu moments and MDM. In [18, 20], contour properties are investigated utilizing a CSS representation. Potential teeth are explicitly extracted and described and the margin is then classified into a set of inferred shape classes. These descriptors are combined base and apex shape descriptors. Cerutti et al. [21] introduces a sequence representation of leaf margins where teeth are viewed as symbols of a multivariate real valued alphabet. In all five studies [18, 20, 21, 73, 85] combining shape and margin features improved classification results in contrast to analyzing the features separately.

**Table 10:** Studies analyzing leaf-specific features either solely or in combination with other leaf features

Organ	Feature	Leaf-specific descriptor	Studies
Leaf	**Vein**	Run-length features	[53]
		Leaf vein and areoles morphology	[78–80]
	Shape, **vein**	Graph representations of veins	[101]
		$$A_{vein}/ A_{leaf}$$	[5, 144]
		Calculating the density of end points and branch points	[107, 108]
		FracDim	[14, 65, 67]
		SC,SIFT	[139]
		Extended circular covariance histogram	[4]
	Color, shape, texture, **vein**	$A_{vein}/ A_{leaf}$	[43, 48]
	**Margin**	Margin signature	[31]
		Leaf tooth features (total number of leaf teeth, ratio between the number of leaf teeth and the length of the leaf margin expressed in pixels, leaf-sharpness and leaf-obliqueness)	[66]
		SC-based descriptors: leaf contour, spatial correlation between salient points of the leaf and its margin	[93]
	Shape **margin**	CSS	[18, 20]
		Sequence representation of leaf margins where teeth are viewed as symbols of a multivariate real valued alphabet	[21]
		Morphological properties of margin shape (13 attributes)	[85]
		Margin statistics (average peak height, peak height variance, average peak distance and peak distance variance)	[73]

### Comparison of Studies (RQ-4)

The discussion of studied features in the previous section illustrates the richness of approaches proposed by the primary studies. Different experimental designs among many studies in terms of studied species, studied features, studied descriptors, and studied classifiers make it very difficult to compare results and the proposed approaches themselves. For this section, we selected primary studies that utilize the same dataset and present a comparison of their results. We start the comparison with the Swedish leaf dataset (Table 11), followed by the ICL dataset (Table 12), and the Flavia dataset (Table 13). A comparison of the other introduced datasets, i.e., ImageCLEF and LeafSnap is not feasible since authors used varying subsets of these datasets for their evaluations making comparison of results impossible.

Classification accuracy as typically reported in studies is defined as follows:

1 $$\begin{aligned} \textit{Accuracy} = \frac{\textit{No.\,of\,correctly\,classified\,images}}{\textit{Total\,No.\,of\,testing\,images}} \times 100 \end{aligned}$$

#### Swedish Leaf Dataset

**Table 11:** Comparison of classification accuracy on the Swedish leaf dataset containing twelve species

Descriptor	Feature	Classifier	Accuracy	Studies
GF	Texture	Fuzzy k-NN	85.75	[136]
FD	Shape	1-NN	87.54	[134, 135]
SC	Shape	k-NN	88.12	[83]
FD	Shape	k-NN	89.60 (83.60)	[62, 147]
HoCS	shape	Fuzzy k-NN	89.35	[136]
TAR	Shape	k-NN	90.40	[94]
HOG	Shape	1-NN	93.17 (92.98)	[145]
MDM–ID	Shape	k-NN	93.60 (90.80)	[62]
IDSC	Shape	1-NN	93.73 (85.07)	[145]
IDSC	Shape	SVM	93.73	[121]
IDSC	Shape	k-NN	94.13 (85.07)	[62]
TOA	Shape	k-NN	95.20	[94]
TSL	Shape	k-NN	95.73	[94]
TSLA	Shape	k-NN	96.53	[94]
LBP	Shape	SVM	96.67	[121]
I-IDSC	Shape	1-NN	97.07	[158]
MARCH	Shape	1-NN	97.33	[135]
DS-LBP	Shape + texture	Fuzzy k-NN	99.25	[136]

The original images of the Swedish leaf dataset contain leafstalks. Numbers in brackets are results obtained after removing leafstalks

*Classifiers.* For the Swedish leaf dataset, nearly all authors apply a *k-nearest neighbor (k-NN)* classifier [62, 62, 83, 94, 136, 147], occasionally in the simple *1-NN* form [134, 135, 145, 158], to perform classification and to evaluate their approaches (see Table 11). k-NN is a non-parametric classification algorithm that classifies unknown samples based to their k nearest neighbors among the training samples. The most frequent class among these k neighbors is chosen as the class for the sample to be classified. A challenge of k-NN is to select an appropriate value of k, typically based on error rates [16]. In order to improve robustness and discriminability of classification, a fuzzy k-nearest neighbors classifier was proposed [136]. Unlike the conventional k-NN, which only considers the congeneric number of k-nearest neighbors, *fuzzy k-NN* synthetically considers the congeneric number and the similarity between the k-nearest neighbors and the unknown sample. Only one study used *support vector machines (SVM)* as classifier on this dataset. A Radial basis function (RBF) kernel for the SVM was used [121], which can handle a high dimensional space of data points that are not linearly separable. SVM are known as classifiers with simple structure and comparatively fast training phase and are easy to implement.

*Classification accuracies.* Table 11 shows classification accuracies achieved on the Swedish leaf dataset with the different methods proposed in the primary studies. The four lowest classification rates are obtained with Gabor Filter (GF) (85.75%), Shape Context (SC) (88.12%), and Fourier descriptor (FD) (87.54 and 89.60%) classified using fuzzy-k-NN, k-NN, and 1-NN. As discussed in the feature section, [94] found TSLA to give better identification scores than TAR, TOA, and TSL. Xiao et al. [145] noticed that the IDSC descriptor performs better than HOG on the original Swedish leaf dataset. Ren et al. [121] used multi-scale overlapped block local binary pattern (LBP) with a SVM classifier and obtained the fourth best classification performance on this dataset. Zhao et al. [158] introduced I-IDSC and obtained with 97.07% the third best result. The multi-scale-arch-height descriptor (MARCH) method [135] achieved the second best classification rate (97.33%). The best result with 99.25% was obtained by [136]. They used dual-scale decomposition and local binary descriptors (DS-LPB). DS-LBP combines textures and contour information of a leaf and is invariant to translation and rotation.

Images of the Swedish leaf dataset contain leafstalks. The benefit of leafstalks is controversially debated by authors. On one hand, they can provide discriminant information for classification, but on the other hand length and orientation of leafstalks depends on the collection and imaging process and is therefore considered unreliable. Table 11 shows that for all with and without leafstalks studied descriptors the classifications accuracy dropped when removing leafstalks, e.g., the performance of IDSC decreased from 93.73 to 85.07%. This result indicates that leafstalks indeed provide useful information for recognition.

#### ICL Dataset

Table 12 shows classification accuracies on the ICL leaf dataset using the methods proposed in the primary studies. The upper part of the table shows results gained on the whole dataset containing 220 species. Several studies do not use the whole dataset, but merely evaluate their approaches on two subsets of the ICL leaf dataset (subset A and B). Subset A includes 50 species sharing the characteristic that the contained species’ shapes can be distinguished easily by humans. Subset B also includes 50 species with shapes that are very similar but still distinguishable [62, 121, 145, 158]. Furthermore, [147, 156, 157] also used a subset of the ICL dataset but without specifying the selected species. Their results are not considered for comparison here.

*Classifier.* The set of utilized classification methods (k-NN, 1-NN, fuzzy k-NN, and SVM) is the same as for the Swedish leaf dataset.

*Classification accuracies.* On the entire dataset, the lowest classification accuracies were obtained with FD, followed by TAR, and IDSC with a simple 1-NN classifier. Similar to the Swedish leaf dataset, the best results were obtained by combining texture and shape features. Wang et al. [140] combined entropy sequence (EnS) representing texture features and center distance sequence (CDS) representing shape features and utilized SVM with a RBF kernel for classification. They achieved the second best classification accuracy with 95.87%. As for the Swedish leaf dataset, the best results were also obtained by [136] using a dual-scale decomposition and local binary descriptors (DS-LPB) and a fuzzy k-NN classifier. Furthermore, classification accuracies of the same methods applied to the Swedish leaf and the ICL dataset show lower accuracies on the ICL dataset, suggesting that species and samples in the ICL leaf dataset represent a more complicated classification task. Wang et al. [136] argues that the ICL dataset contains many species with similar shapes. This characteristic can also explain a higher drop in classification accuracies for shape-based methods, such as HoCS, IDCS, and MDM, than for texture-based methods. A similar effect is visible for the subsets A and B containing 50 species. Subset B (accuracies in brackets) consistently yields lower accuracies than subset A. Especially, IDCS is found to be not a discriminative descriptor for distinguishing leaves with visually similar shapes, it obtains an accuracy of only 64% on subset B compared to 96% on subset A.

**Table 12:** Comparison of classification accuracies on the ICL dataset (220 species) and its two subsets (50 species each)

Descriptor	Feature	Classifier	Accuracy	Studies
*Full dataset: 220 species*				
FD	Shape	1-NN	60.08	[135]
TAR	Shape	1-NN	78.25	[135]
IDCS	Shape	1-NN	81.39	[135]
IDSC	Shape	k-nn	83.79	[139]
GF	Texture	Fuzzy k-NN	84.60	[136]
MARCH	Shape	1-NN	86.03	[135]
HoCS	Shape	Fuzzy k-NN	86.27	[136]
MDM	Shape	Fuzzy k-NN	88.24	[136]
IDSC	Shape	Fuzzy k-NN	90.75	[136]
SIFT, SC	Shape + vein	k-NN	91.30	[139]
EnS and CDS	Shape + texture	SVM	95.87	[140]
DS-LBP	Shape + texture	Fuzzy k-NN	98.00	[136]
*Subsets: 50 species*				
IDSC	Shape	SVM	95.79 (63.99)	[121]
FD	Shape	1-NN	96.00 (80.88)	[62]
HOG	Shape	SVM	96.63 (83.35)	[121]
LBP	Shape	SVM	97.70 (92.80)	[121]
IDSC	Shape	1-NN	98.00 (66.64)	[62, 145]
MDM with ID	Shape	1-NN	98.20 (80.80)	[62]
HOG	Shape	1-NN	98.92 (89.40)	[145]
I-IDSC	Shape	1-NN	99.48 (88.40)	[158]

Certain studies used two subsets of the ICL leaf dataset: subset A and subset B (in brakets). Subset A includes 50 species with shapes easily distinguishable by humans. Subset B includes 50 species with very similar but still visually distinguishable shapes

#### Flavia Dataset

The Flavia dataset is a benchmark used by researchers to compare and evaluate methods across studies and publications. The dataset contains leaf images of 32 different species. Table 13 shows a comparison of different methods applied by the primary studies on the Flavia dataset.

*Classifier.* Primary studies used a richer set of classification methods for their experiments on the Flavia dataset compared to the Swedish leaf dataset and the ICL dataset. In addition to the previously mentioned k-NN and SVM classifiers, also the following methods were used: Naive Bayes (NB) [16], decision tree (DT) [116], random forest (RF) [16], neuro fuzzy classifier (NFC) [23, 24], multi-layered perceptron (MLP) [23], Riemannian metrics [77], artificial neural network (ANN) with back-propagation (BPNN) [1], and probabilistic neural networks (PNN) [58, 144]. *Bayesian classifiers* are statistical models able to predict the probability for an unknown sample to belong to a specific class. They are a practical learning approach based on Bayes’ Theorem. A disadvantage of Bayesian classifiers is that conditional independence may decrease accuracy thereby imposing a constraint over attributes that may not be dependent. A *Decision Tree* is a classifier that uses a tree-like graph to represent decisions and their possible consequences. A decision tree consists of three types of nodes: decision nodes, which evaluate each feature at a time according their relevance; chance nodes, which choose between possible values of features; and end nodes, which represent the final decision, i.e., the wing label. The *Random Forest* classifier is based on the classification tree approach. It aggregates predictions of multiple classification trees for a dataset. Each tree in the forest is grown using bootstrap samples. At prediction time, classification results are taken from each tree in the forest. The class with the most votes among the separate trees is selected by the forest. Random forests are efficient on large datasets with high accuracy. Random forests also allow to estimate the importance of input variables (in their original dimensional space). However, they have constraints on memory and computing time. Finally, an *artificial neural network (ANN)* is an interconnected group of artificial neurons simulating the thinking process of the human brain. One can consider an ANN as a “magical” black box trained to achieve an expected intelligent process, against the input and output information stream [144].

*Classification accuracies.* The lowest classification rates with 25.30% were obtained with Hu moments [111] and Hu moments in combination with curvelet transform 41.6% [23]. The results demonstrate that the Hu descriptor is not robust when working with leaf shape and should be combined with other features like vein, margin, color, or texture [23, 111]. Prasad et al. [116] study shape and color information of leaves using SMSD and FD to represent the shape. Once the initial classification is calculated solely based on these shape descriptors using k-NN, the two classes with the highest probability are selected. Then, color is analyzed and a binary decision tree is used to decide between these two classes. Prasad et al. [116] found that color information of leaves increased accuracy from 84.45% (shape only) to 91.30% (shape + color). Arun Priya et al. [5] compared SVM with RBF kernel and k-NN classification based on shape and vein features and found that SVM with 94.5% outperformed k-NN with only 78%. Caglayan et al. [16] compared four classification algorithms: k-NN, SVM with linear kernel function, Naive Bayes, and Random Forest based on shape and color features. Across all their experiments, Random Forest yielded the best classification results. The lowest accuracy was achieved with SVM based on shape features. Combining shape and color increased classification accuracy significantly. The greatest increase was demonstrated with SVM using SMSD, color moments, and color histograms improving accuracy about 15% compared to a Naive Bayes classifier using the same features. Wang et al. [140] obtained the highest accuracy on the Flavia dataset with 97.80% by combining EnS representing texture features with CDS representing shape features and utilized SVM with RBF kernel for classification (see results of ICL dataset).

Four primary studies used neural network classifiers [1, 23, 58, 144]. Aakif and Khan [1] applied back-propagation neural networks (BPNN) and obtained a classification accuracy of 96.0%. Hossain and Amin [58] and Wu et al. [144] applied probabilistic neural networks (PNN) for classification of leaf shape features and obtained an accuracy of 90.31 and 91.40% respectively. The PNN learns rapidly compared to the traditional back-propagation, and guarantees to converge to a Bayes classifier if enough training examples are provided, it also enables faster incremental training and is robust to noisy training samples [58]. Chaki et al. [23] used two types of supervised feed-forward neural classifiers: a multi-layered perceptron using back propagation (MLP) and a neuro fuzzy classifier using a scaled conjugate gradient algorithm (NFC). The accuracies obtained by solely using texture-based descriptors are 81.6% with NFC and 87.1% with MLP, by only using shape-based descriptors a significantly lower accuracy of 50.16% using NFC and 41.6% using MLP were obtained. As for the Swedish leaf dataset and the ICL dataset, the combination of texture and shape obtained the best results. Chaki et al. [23] found that by combining texture and shape, classification accuracy rose to 97.6% with NFC and dropped to 85.6% with MLP. The former being the second highest accuracy achieved on the Flavia dataset.

**Table 13:** Comparison of classification accuracies on the FLAVIA dataset with 32 species

Descriptor	Feature	Classifier	Accuracy	Study
Hu moments	Shape	SVM	25.30	[111]
HOG	Shape		**84.70**	
SIFT	Shape		87.50	[81]
SMSD, $A_{vein}/ A_{leaf}$	Shape + vein	PNN	90.31	[144]
SMSD	Shape		70.09	
PFT	Shape	k-NN	76.69	[116]
SMSD, FD	Shape		84.45	
SMSD, FD, CM	Color + shape	k-NN, DT	**91.30**	
SMSD	Shape	PNN	91.40	[58]
SMSD, $A_{vein}/ A_{leaf}$	Shape + vein	SVM (k-NN)	**94.50** (78.00)	[5]
SIFT	Shape	SVM	95.47	[59]
SURF	Shape	SVM	95.94	[103]
SMSD, FD	Shape	BPNN	96.00	[1]
SMSD, CM, GLCM, $A_{vein}/ A_{leaf}$	Shape + color + texture + vein	SVM	96.25	[48]
SMSD	Shape		87.61 (82.34, 80.26, 72.89)	[16]
SMSD, CM	Shape + color	RF (k-NN, NB, SVM)	93.95 (92.46, 88.77, 86.50)	
SMSD, CM, CH	Shape + color		**96.30** (94.21, 89.25, 92.89)	
SMSD	Shape	NFC	97.50	[24]
CT, Hu moments	Shape		50.16 (41.60)	[23]
GF, GLCM	Texture	NFC (MLP)	81.60 (87.10)	
CT, Hu moments, GF, GLCM	Shape + texture		**97.60** (85.60)	
EnS and CDS	Shape + texture	SVM	**97.80**	[140]

### Prototypical Implementation (RQ-5)

In addition to studying classification approaches, 13 studies provide an implementation of the proposed method as app for mobile devices [11, 20, 26, 76, 87, 100, 101, 103, 111, 112, 116, 134, 135], two studies as a web service [68, 110], and four studies as a desktop application [57, 58, 102, 158].

*Mobile applications.* A smartphone possesses everything required for the implementation of a mobile plant identification system, including a camera, a processor, a user interface, and an internet connection. These preconditions make smartphones highly suitable for field use by professionals and the general public. However, these devices still have less available memory, storage capacity, network bandwidth and computational power than desktop or server machines, which limits algorithmic choices. Due to these constraints, it can be tempting to offload some of the processing to a high performance server. This requires a reliable internet connection (Table 14). Using an online service can be attractive when dataset or algorithm are likely to be updated regularly or when they have large computational and memory requirements. However, in remote areas where plant identification applications are likely to be most useful, an internet connection may be unreliable or unavailable. The contrary approach is using efficient algorithms that run directly on the device without the need for a network connection or a support server but with potential limitations in their classification performance [134].

Belhumeur et al. [11] developed LeafView, a Tablet-PC based application for the automated identification of species in the field. Leaf images are captured on a plain background. A computer vision component finds the best set of matching species and results are presented in a zoomable user interface. Samples are matched with existing species or marked unknown for further study. LeafView was built with C#, MatLab, and Piccolo. Kumar et al. [76] designed Leafsnap, the so far most popular mobile app based on iOS for plant species identification. A user can take a photo of a leaf on plain background, transfer the image to the Leafsnap server for analysis, and eventually see information about the identified species. This application is restricted to tree species of the Northeastern United States and can perform the identification only with access to the internet. Cerutti et al. [20] provide an educational iOS application called FOLIA to help users recognizing a plant species in its natural environment. In order to perform this task, the application first lets the user take a picture of a unknown leaf with the smartphone camera. Then, it extracts high-level morphological features to predict a list of the most corresponding species.

Ma et al. [87] implemented an Android-based plant image retrieval system in JAVA. Here the user is supposed to place a single leaf taken on a light, untextured, and uniform background. Compared to [76], users can identify the species without internet and also use existing digital images as query image, i.e., for identifying a species. Also [134, 135] implemented an Android application in Java. Classification can alternatively be performed on the server for more computationally expensive algorithms or offline on the device. Even in online mode, only a feature vector is being sent to the server rather than the actual image. The feature extraction is performed on the device thereby drastically reducing bandwidth requirements for the server connection. The server returns a dynamic webpage, opened in the device’s browser, showing closest matches. Another Android application has been developed by [103]. Similar to Leafsnap, this system uses a client-server implementation. Initially, a user takes a leaf photo with the phone. This photo is then being sent to the server on which it is analyzed in order to identify the species. The server procedure contains of two main analyzes. First, a leaf/no-leaf classification aims at checking the validity of the uploaded photo. Second, for leaf containing photos the species identification is triggered, otherwise the system will ask for another photo. Upon a leaf identification, the client will display species information to the user. Chathura Priyankara and Withanage [26] developed an Android client application, which interacts with a leaf recognition algorithm running on the server through a SOAP-based web service. OpenCV is used for the actual image processing. Prasad et al. [116] developed an offline mobile application for Android using OpenCV. Leaf images are captured with the device’s camera and must exhibit a uniform background for simplifying the segmentation. The classification process is done on the mobile device.

*Web services.* Pauwels et al. [110] implemented a web service that allows users to upload a tree leaf image. The service is designed as a two-tier system. The front-end allows to upload query images and the back-end performs the matching. Eventually, a webpage is created showing the ten most similar exemplars along with the names of the species. Pham et al. [111] developed among others a graphical web tool of their approach. This version is developed in PHP and uses a mySQL database. Joly et al. [68] developed Pl@ntNet Identify, an interactive web service dedicated to the content-based identification of plants using general public contributed image data. It is composed of three main parts: an interactive web GUI for the client, a content-based visual search engine, and a multi-view fusion module on the server side. Pl@ntNet Identify was the first botanical identification system able to consider a combination of habit, leaf, flower, fruit, and bark images for classification. In the meantime, Pl@ntNet also provides a mobile version of their service on iOS and Android.

*Desktop applications.* Hossain and Amin [58] developed the Chloris desktop application for plant identification. The system was trained with 1200 images of simple leaves on plain background from 30 plant species. They also tested their system with partially damaged leaves and demonstrated that it was able to successfully identify the plants. However, no more information about the system is given. Hong and Choi [57] implemented a flower recognition system with Microsoft Visual Studio to evaluate the performance of their proposed recognition process. Based on a flower image, the system finds the contour of flowers using color and edge information and then extracts image features of flowers. The system compares these features with the features of images stored in the system. Eventually, the system determines species with the most similar features and presents the top three ranked species.

**Table 14:** Prototypical applications implementing proposed approaches

Name	Application type	Organ	Background	Analysis	URL	Studies
LeafView	Mobile (Tablet PC)	Single leaf	Plain	Offline		[11]
LeafSnap	Mobile (iOS)	Single leaf	Plain	Online	http://leafsnap.com/	[76]
FOLIA	Mobile (iOS)	Single leaf	Natural	Online	https://itunes.apple.com/app/folia/id547650203	[20]
ApLeafis	Mobile (Android)	Single leaf	Plain	Offline		[87]
–	Mobile (Android)	Single leaf	Plain	Online		[103]
–	Mobile (Android)	Single leaf	Plain	Offline		[116]
–	Mobile (Android)	Single leaf	Plain	Offline/online		[134, 135]
–	Mobile (Android)	Single leaf	Plain	Online		[26]
–	Mobile (iOS) + web	Single leaf	Plain	Offline/online		[111]
CLOVER	Mobile (PDA)	Single leaf	Plain	Online		[100, 101]
MOSIR	Mobile	Flower	Natural	Online		[112]
Leaves Lite	Web	Single leaf	Plain	Online		[110]
Pl@ntNet-Identify	Web	Multi organ	Plain	Online	http://identify.plantnet-project.org/	[68]
Chloris	Desktop	Single leaf	Plain	Offline		[58]
Leaf recognition	Desktop	Single leaf	Plain	Offline		[158]
–	Desktop	Single leaf	Natural	Offline		[102]
Flower recognition system	Desktop	Flower	Natural	Offline		[57]

## Discussion

This paper aimed at identifying, analyzing, and comparing research work in the field of plant species identification using computer vision techniques. A systematic review was conducted driven by research questions and using a well-defined process for data extraction and analysis. The following findings summarize principal results of this systematic review and provide directions for future research.

### Finding-1: Most studies conducted by computer scientist

Automated plant species identification is a topic mostly driven by academics specialized in computer vision, machine learning, and multimedia information retrieval. Only a few studies are conducted by interdisciplinary groups of biologist and computer scientists. Increasingly, research is moving towards more interdisciplinary endeavors. Effective collaboration between people from different disciplines and backgrounds is necessary to gain the benefits of joined research activities and to develop widely accepted approaches [13]. This is also the case for automated plant species identification. Here biologist can learn from computer science methods and vice versa. For example, leaf shape is very important not only for species identification, but also in other studies, such as plant ecology and physiology. We therefore foresee an increasing interest in this trans-disciplinary challenge.

### Finding-2: Only two approaches evaluated on large datasets

Since there exist more than 220,000 plant species around the world [52, 90, 125], it is important to develop plant identification methods capable of handling this high variability. Only two primary studies evaluated their approaches on large datasets with realistic numbers of species [68, 143]. Furthermore, considering that changes in illumination, background, and position of plants or their organs may create dramatically different images for the same plant, also larger datasets in this regard are required to yield high accuracy in plant identification under realistic conditions. Apart from the effort for acquiring the required images, further research is necessary to effectively store, handle, and analyze such large numbers of images [143].

### Finding-3: Most studies used images with plain background avoiding segmentation

Most analyzed images in the studies were taken under simplified conditions (e.g., one mature leaf per image on plain background). If the object of interest is imaged against a plain background, the often necessary segmentation in order to distinguish foreground and background can be performed fully automated with high accuracy. Segmenting the leaf with natural background is particularly difficult when the background shows a significant amount of overlapping green elements. Towards real-life application, studies should utilize more realistic images containing multiple leafs, having a complex background, and been taken in different lighting conditions.

### Finding-4: Main research focus on leaf analysis for plant identification

Except for one study [68], proposed approaches for plant identification are based on the analysis of only one of the plant’s organs. The most widely studied organs are leaf followed by flower. Reasons for focusing on leaves in plant identification are that leaves are available for examination throughout most of the year, that they are easy to find and to collect, and that they can easily be imaged compared to other plant morphological structures, such as flowers, barks, or fruits [33]. These characteristics simplify the data acquisition process. In contrast, traditional keys often utilize flowers or their parts to characterize species, but flowers are typically only available for a few weeks of the year during the blooming season. A smaller number of 13 primary studies proposed to identify species solely based on flowers. Researchers even argue [29] that machine learning based flower classification is one of the most difficult tasks in computer vision. If captured in their habitat, images of flowers greatly vary due to lighting conditions, time, date, and weather. Due to being a complex 3D object, there is also variation in viewpoint, occlusions, and scale of flower images compared to leaf images. All these problems make flower-based classification a challenging task. On the positive side, the segmentation of typically colored flowers in their natural habitat can be considered an easier task than the segmentation of leaves in the same setting.

### Finding-5: Shape is the dominant feature for plant identification

Shape analysis of leaves has received by far the most attention among the primary studies. Leaf shape is considered more heritable and often favored over leaf geometry since this is largely influenced by a plant’s habitat. Although species’ leaves differ in detail, differences across species are often obvious to humans. Most text-based taxonomic keys involve leaf shape for discrimination. Additionally, leave shape is among the easiest aspect for automated extraction assuming that the leaf can easily be separated from a plain background. Shape analysis of flowers has also been considered for species identification. For example, the shape of individual petals, their configuration, and the overall shape of a flower can be used to distinguish between flowers and eventually species. However, the petals are often soft and flexible making them bend, curl, or twist; which lets the shape of the same flower appear very different. The difficulty of describing the shape of flowers is increased by natural deformations. Furthermore, a flower’s shape typically also changes with its age to the extent where petals even fall off [104].

### Finding-6: Multi-feature fusion facilitates higher classification accuracy

Several primary studies showed the benefits of multi-feature fusion in terms of a gain in classification accuracy [10, 16, 23, 65, 116]. Although texture is often overshadowed by shape as the dominant or more discriminative feature for leaf and flower classification, it is nevertheless of high significance as it provides complementary information. This review revealed that texture is the feature that highly influenced the identification rate. In particular, texture captures leaf venation information as well as any eventual directional characteristics, and more generally allows describing fine nuances or micro-texture at the leaf or flower surface [148]. Color is not expected to be as discriminative as shape or texture for leaf analysis, since most leaves are colored in some shade of green that also vary greatly under different illumination [148]. In addition to the low inter-class variability in terms of color, there is also high intra-class variability, i.e., even the colors of leaves belonging to the same species or even plant can present a wide range of colors depending on the season and the plant’s overall condition (e.g., nutrient and water). For example, many dried leaves turn brown, so color is not usually a useful feature for leaf analysis. Regardless of the aforementioned complications, color can still contribute to plant identification, considering leaves that exhibit an extraordinary hue [148]. However, further investigation on leaf color is necessary. For flower analysis color plays a more important role. Color as feature is also known for its low dimensionality and low computational complexity thus making it convenient for real-time applications. Despite being a useful feature of leaves in traditional species identification, leaf margin has seen little use in automated species identification being studied by only 8 out of 106 studies. Reasons may be that teeth are not present for all plant species, that teeth can easily be damaged or get lost before and after specimen collection, and that it is difficult to acquire quantitative margin measurements automatically [34]. Also vein structure as a leaf-specific feature plays a subordinate role and should be explored more deeply in the future.

### Finding-7: Contour-based shape description more popular than region-based description

Research on contour-based shape description is more active than that on region-based shape description. A possible explanation is that humans discriminate shapes mainly by their contour features. A major difficulty for contour-based methods is the problem of ’self-intersection’. This is where part of a leaf overlaps other parts of the same leaf and can result in errors when tracing the outline. Self-intersection occurs especially with lobed leaves, and may not even occur consistently for a particular species. Furthermore, the performance of contour-based approaches is often sensitive to the quality of the contour extracted in a segmentation process, which naturally complicates distinguishing between species with very similar shapes. However, region-based methods are more robust as they use the entire shape information. These methods can cope well with shape defection which arises due to missing shape part or occlusion.

### Finding-8: Cross-comparing and evaluating proposed methods is very difficult

For the analysis of experimental results, researchers use different datasets, the size of samples is different per dataset as well as the reported evaluation metrics (e.g., rank-1 accuracy, rank-10 accuracy, precision). This makes it difficult to compare the performance of different approaches. Efficient evaluation criteria are necessary for plant recognition. Using the same evaluation criteria makes the evaluation of proposed methods more objectively.

### Finding-9: LeafSnap, Pl@ntNet, and Folia only publicly available implementations

Some of the proposed approaches have been implemented as web, mobile, or desktop application and have initiated interactions between computer scientists and end-users, such as ecologists, botanists, educators, land managers, and the general public [71]. Mobile applications offering image-based identification services are particularly promising for setting-up massive ecological monitoring systems, involving many contributors at low cost. One of the first system in this domain was the LeafSnap application (iOS), supporting a few hundred tree species of North America. This was followed by other applications, such as Pl@ntNet (iOS, Android, and web) and Folia (iOS) dedicated to the European flora [70]. As promising as these applications are, their performances are still far from the requirements of a real-world social-based ecological surveillance scenario. Allowing the mass of citizens to produce accurate plant observations requires to equip them with much more accurate identification tools [50].
